# Irisin mediates beiging of adipose-derived mesenchymal stem cells through binding to TRPC3

**DOI:** 10.1186/s12915-022-01287-2

**Published:** 2022-05-02

**Authors:** Chunling Xue, Xuechun Li, Li Ba, Yamei Shen, Zhao Sun, Junjie Gu, Ying Yang, Qin Han, Robert Chunhua Zhao

**Affiliations:** 1 Institute of Basic Medical Sciences Chinese Academy of Medical Sciences, School of Basic Medicine Peking Union Medical College, Peking Union Medical College Hospital, Center of Excellence in Tissue Engineering Chinese Academy of Medical Sciences, Beijing Key Laboratory (No.BZO381), Beijing, China; 2 Department of oncology, Peking Union Medical College Hospital, Chinese Academy of Medical Science and Peking Union Medical College, No. 1 Shuaifuyuan Hutong, Dongcheng District, Beijing, 100730 People’s Republic of China

**Keywords:** Mesenchymal stem cells, Beiging, IRISIN, TRPC3, Calcium influx, Energy metabolism

## Abstract

**Background:**

Beiging of white fat plays an important role in energy metabolism. Beige adipocytes contribute to the regulation of body weight and body temperature through expenditure of chemical energy to produce heat, and they have therefore recently attracted considerable attention as potential targets for therapeutic approaches in metabolic disorders, including obesity. All adipocytes, including beige adipocytes, differentiate from mesenchymal stem cells (MSCs), which may provide an important path for clinical intervention; however, the mechanism of beiging of human adipose cell-derived MSCs is not fully understood. Here, we provide insights on the role of IRISIN, which is known to be secreted by skeletal muscle and promote beiging of white fat.

**Results:**

We established an IRISIN-induced mesenchymal stem cell beiging model and found that IRISIN protein interacts with the MSC membrane protein TRPC3. This interaction results in calcium influx and consequential activation of Erk and Akt signaling pathways, which causes phosphorylation of PPARγ. The phosphorylated PPARγ enters the nucleus and binds the UCP1 promoter region. Furthermore, the role of TRPC3 in the beiging of MSCs was largely abolished in Trpc3^−/−^ mice. We additionally demonstrate that the calcium concentration in the brain of mice increases upon IRISIN stimulation, followed by an increase in the content of excitatory amino acids and norepinephrine, while Trpc3^−/−^ mice exhibit the reverse effect.

**Conclusions:**

We found that TRPC3 is a key factor in irisin-induced beiging of MSCs, which may provide a new target pathway in addressing metabolic disorders. Our results additionally suggest that the interaction of irisin with TRPC3 may affect multiple tissues, including the brain.

**Graphical abstract:**

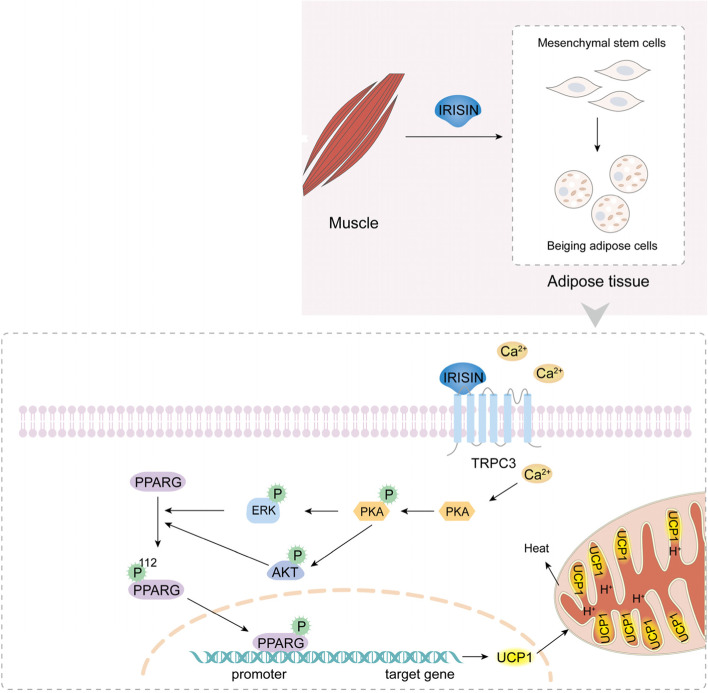

**Supplementary Information:**

The online version contains supplementary material available at 10.1186/s12915-022-01287-2.

## Background

Obesity is a major risk factor for many metabolic disorders including type 2 diabetes (T2D), cardiovascular disease, and several types of cancer [1–3]. Recently, a type of fat cells, called beige fat cells, have attracted attention as the beige fat can dissipate calories as heat, in the hope of activating the process to combat obesity. Beige fat cells can be a potential new therapeutic target for the metabolic disorders [4–7]. In mammals, there are three types of adipose tissue: white adipose tissue, brown adipose tissue, and beige adipose tissue. White adipose tissue accounts for more than 10% of the body weight of a normal healthy individual and is specialized to store excess energy. Brown fat and beige fat can release ATP in the form of heat which play an important role in regulating body weight and body temperature [8, 9]. Typical brown fat cells and skeletal muscle cells originate from the muscle layer of the dermis and dermomyotome cells, which are localized significantly in dedicated BAT depots, whereas beige/brite cells originate postnatally from the large white endothelial cells and perivascular cells [8, 10–12]. The main function of brown and beige fat is to prevent hypothermia, especially in infants, young mammals, and hibernating animals. Beige adipocytes have the inducible nature which indicates their relevance to adult humans. When we do not know whether subjects possess appreciable levels of existing BAT especially including obese, diabetic, and elderly individuals, therefore promoting the beiging of white fat may be beneficial for the treatment of obesity and type 2 diabetes [8, 13]. Beige fat cells generate heat through the uncoupling protein 1 (UCP1) located on the intimal surface of the mitochondria. Upon activation, UCP1 catalyzes the movement of protons across the mitochondrial membrane, resulting in the uncoupling of oxidative phosphorylation during ATP synthesis [14, 15]. Further study of these mechanisms will allow us to easily control thermogenic organs and may provide a new therapeutic method for obesity and obesity-related diseases.

Mesenchymal stem cells (MSCs) exhibit multi-directional differentiation potential and play an important role in tissue regeneration [16–18]. Previous studies have indicated that MSCs are present in almost all tissues and can be easily isolated from various tissues [19, 20]. At present, researchers have found that white adipose tissue (WAT) contained a putative MSC population in human which can promote its differentiation into adipocytes, osteoblasts, chondrocytes, and myoblasts [21]. The role of MSCs in beige of adipose tissue is well worth studying. It has been reported that IRISIN can induce MSC beige, but its mechanism is not clear. The role of IRISIN in muscles, cardiovascular tissue, neurons, and energy metabolism has been widely studied. For example, exercise stimulates IRISIN secretion by cleaving the precursor protein, fibronectin III structure containing 5 (FNDC5) [22, 23], which induced the “browning” of subcutaneous white adipose tissues. However, this protein played little effect on the classical brown fat cells that were isolated from the interscapular depot [22, 24]. PPARγ-coactivator 1α(PGC1-α) can promote the synthesis of FNDC5, which is made up of 212 amino acids in humans and 209 amino acids in rat/mouse [22, 25]. Previously, IRISIN was found to promote the browning of white adipocytes and induce UCP1 expression by activating the p38 mitogen-activated protein kinase (MAPK) and extracellular-signal regulated kinase (ERK) signaling pathways [26]. Recent data has shown that IRISIN can also promote the AKT phosphorylation in the H9C2 cells and cardiomyocytes [26]. These signaling pathways are important in cardiac development, remodeling, and metabolism [27, 28]. Kim et al. reported that the functional receptor of IRISIN, αV Integrin, provides a basis for further studies, but the data do not rule out the possibility of other receptors [29]. The concentration of circulating IRISIN in adipose tissue was significantly decreased in obese and type 2 diabetic patients [30]. It has been reported that all adipocytes are differentiated from MSCs in a process known as adipogenesis [30]. In this study, we use IRISIN to stimulate MSCs in order for them to differentiate into beige fat cells and further explores the mechanisms which may reveal a treatment method for obese diseases.

The TRPC superfamily is divided into seven subfamilies, which can be classified into two major subgroups: The TRPC1/4/5 subgroup constitutes store-operated Ca^2+^ channels (SOCs) and transient receptor potential channel, canonical 3/6/7(TRPC3/6/7) constitutes the receptor-operated channels. The TRPC family constitutes ion channel proteins that encode a G-protein-coupled membrane receptor linked to phospholipase C and mediates the function of many cells [31, 32]. TRPC3 is highly expressed in the brain, smooth muscles, and cardiomyocytes [33, 34]. In endothelial cells and smooth muscle cells, TRPC3 is activated by purine receptors, resulting in Ca^2+^, depolarization, and smooth muscle cell vasoconstriction [35, 36]. TRPC3 is also activated by T cell receptors, which mediate the T lymphocyte immune response. The mechanism underlying the TRPC3-mediated immune response involves the direct binding of TRPC3 to PLCγ2 [37–39]. However, the role of TRPC3 in adipose tissue is rarely reported.

In this study, we first used mass spectrometry of protein complexes immunoprecipitated with irisin, which preliminary screened interacting proteins of IRISIN. We found that direct interaction between IRISIN and TRPC3 and how their interaction promote the differentiation of MSCs into beige cells. We developed IRISIN-induced mesenchymal stem cell beiging model to evaluate the role and mechanism of IRISIN and TRPC3 function in beige cell differentiation of adipose tissue-derived MSCs (AD-MSCs).

## Results

### IRISIN promotes the differentiation of MSCs into beige cells

**Fig. 1:**
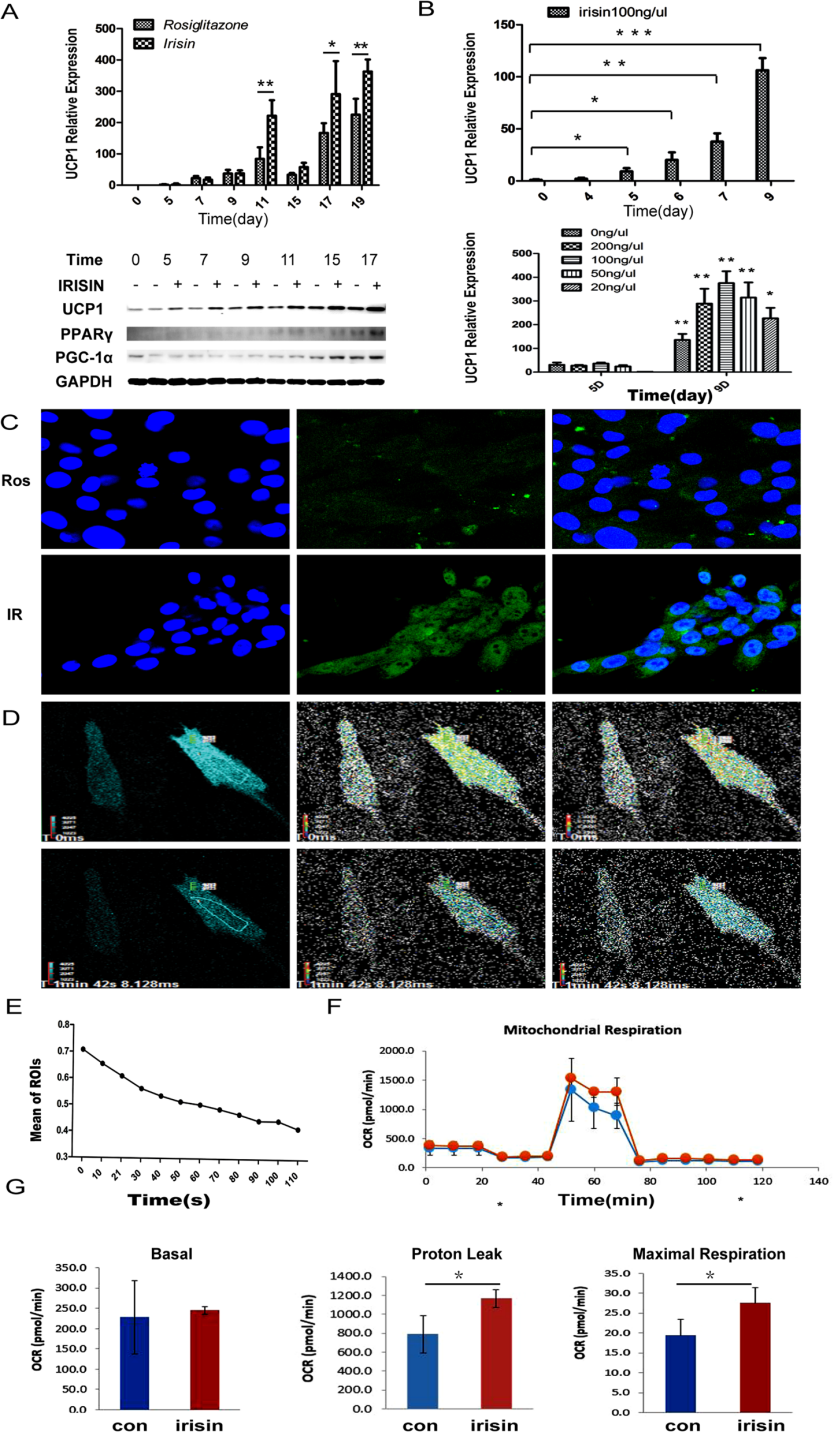
IRISIN induces the differentiation of mesenchymal stem cells into beige fat cells. **A** Quantification of real-time fluorescence for beiging fat cell marker gene *UCP1*(above). UCP1, PPARγ, and PGC-1α protein expression was detected by western blotting (below). **B** Quantification of real-time fluorescence for *UCP1* at different IRISIN stimulation durations (days 0, 4, 5, 6, 7, and 9) (*P* < 0.05) and at different concentrations (0, 20, 50, 100, and 200 ng/μL) of IRISIN. **C** Immunofluorescence assay for analyzing UCP1 expression. **D** Real-time monitoring of ATP by fluorescence microscopy. ATP level changes upon IRISIN stimulation in beige fat cells. **E** Changes in ATP levels at different time points. ATP production was significantly reduced with time. **F, G** Real-time quantification of mitochondrial oxidative respiration rate using Seahorse XF Extracellular Flux Analyzers. Cell mitochondrial respiration rate was significantly enhanced upon IRISIN stimulation .*p < 0.05, **p < 0.01, and ***p < 0.001.

To assess the function of IRISIN in beige cell differentiation of AD-MSCs, we used two groups of cells: the control group induced only with rosiglitazone which induces white fat beiging (abbreviated as Con) and the experimental group co-stimulated with IRISIN protein and rosiglitazone (abbreviated as IR). *UCP1* expression in the IR group was significantly higher than that in the Con group, and WB results showed that UCP1, PPARγ, and PGC-1α expression were increased in the IR group compared to the Con group (Fig. 1A). Notably, *UCP1* levels increased in the IR group after 5 days and remained elevated at 9 days. IRISIN raised the *UCP1* mRNA level in beiging cultures in a dose-dependent manner and the induction efficiency of IR was the highest when 100 ng/μL IRISIN was applied (*P* < 0.05) (Fig. 1B). UCP1 expression in the IR group was higher than that in the rosiglitazone-induced beige cells (Fig. 1C). Beige fat cells are known to consume ATP to release heat. Amount of ATP in the IR group was lower than that in the con group (Fig. 1D, E). Further, we measured the oxygen consumption rate (OCR) in cells treated with IRISIN using Seahorse analyses (Fig. 1F). We found that IRISIN treatment increased the proton leakage and maximum respiratory capacity in primary beige cells with the same basal level at 7 days (*P* < 0.05) (Fig. 1G). Taken together, our data showed that IRISIN could efficiently induce beige differentiation of MSCs.

### IRISIN induces the differentiation of MSCs into beige cells by binding to TRPC3

**Fig. 2:**
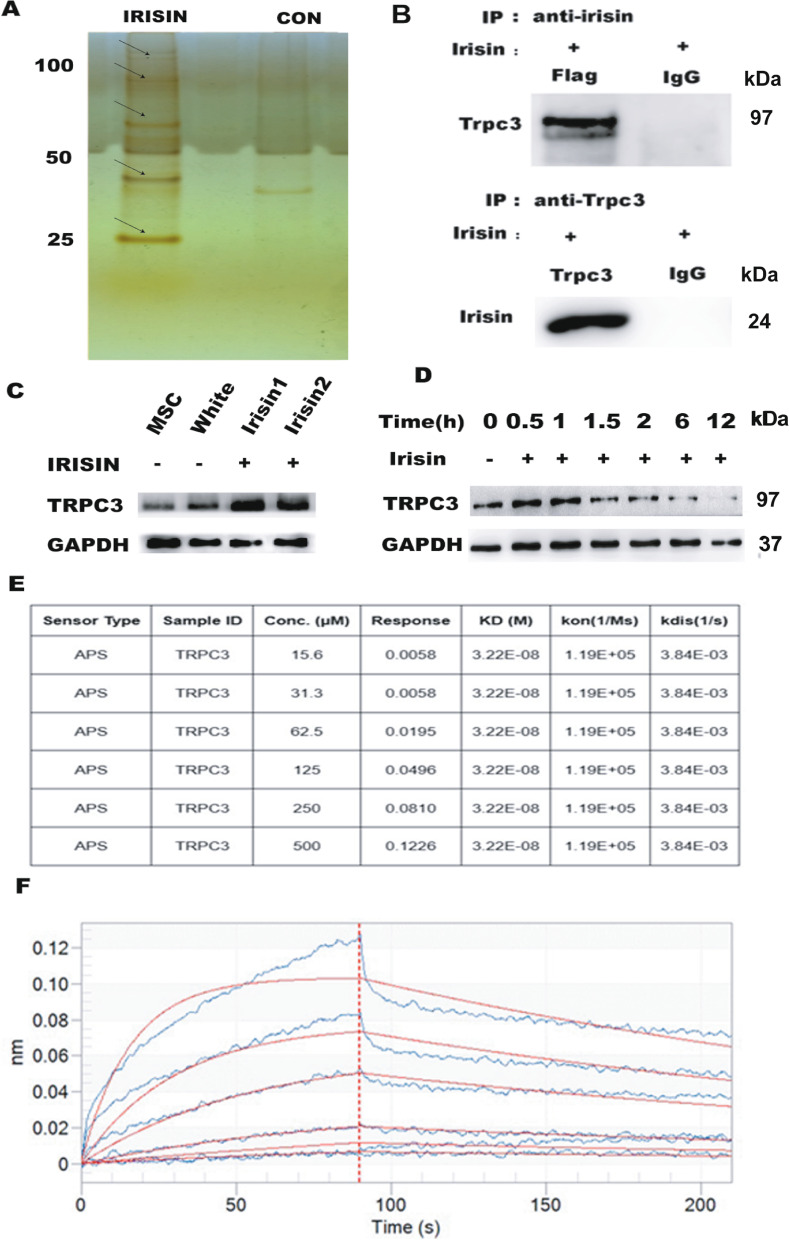
IRISIN promotes beige cell differentiation of mesenchymal stem cells by binding TRPC3. **A** Protein was isolated from the rosiglitazone- and IRISIN-treated cells and subjected to sodium dodecyl sulfate polyacrylamide gel electrophoresis (SDS-PAGE). Protein bands were detected by silver staining. Preliminary screening revealed TRPC3 as an interacting protein. **B** Screening of 293T cells after transfection with IRISIN and TRPC3 plasmids by co-immunoprecipitation. **C** TRPC3 protein expression was detected by western blotting. **D** Western blotting analysis of TRPC3 expression over time. **E, F** The interaction between TRPC3 and IRISIN was detected using biofilm interference (BLI). The data were analyzed using Data Analysis software 9.0

We evaluated the mechanism involved in IRISIN-mediated differentiation of MSCs into beige cells. We performed mass spectrometry of protein complexes immunoprecipitated with IRISIN and preliminary screening of interacting proteins (Fig. 2A, Additional file 6: Table S1). Based on the results obtained, we tested whether IRISIN could bind to TRPC3. We used 293T cells, which do not express the TRPC3 protein, for overexpressing IRISIN and TRPC3 proteins. To our surprise, the results of co-immunoprecipitation assays showed that IRISIN proteins strongly interacted with TRPC3 (Fig. 2B). As expected, the amounts of TRPC3 proteins were increased in beiging adipose cells compared with MSCs and white adipose cells (Fig. 2C). Notably, TRPC3 levels were enhanced in MSCs after 30 min of IRISIN treatment and were reduced at 90 min (Fig. 2D). Biofilm interference was employed to quantify the binding kinetics of IRISIN to TRPC3. The interaction kinetics between the IRISIN and TRPC3 proteins revealed a positive correlation between the signal and concentration of the analyte. (Note: KD represents the strength of the combination, the smaller the number, the stronger the combination. Usually the KD value between proteins ranges 10^−8^–10^−11^ M. If KD > 100 nM (10^−7^ M), the binding capacity between the two proteins was considered to be relatively weak) (Fig. 2E, F).

### IRISIN and TRPC3 activate Ca^2+^ influx

**Fig. 3:**
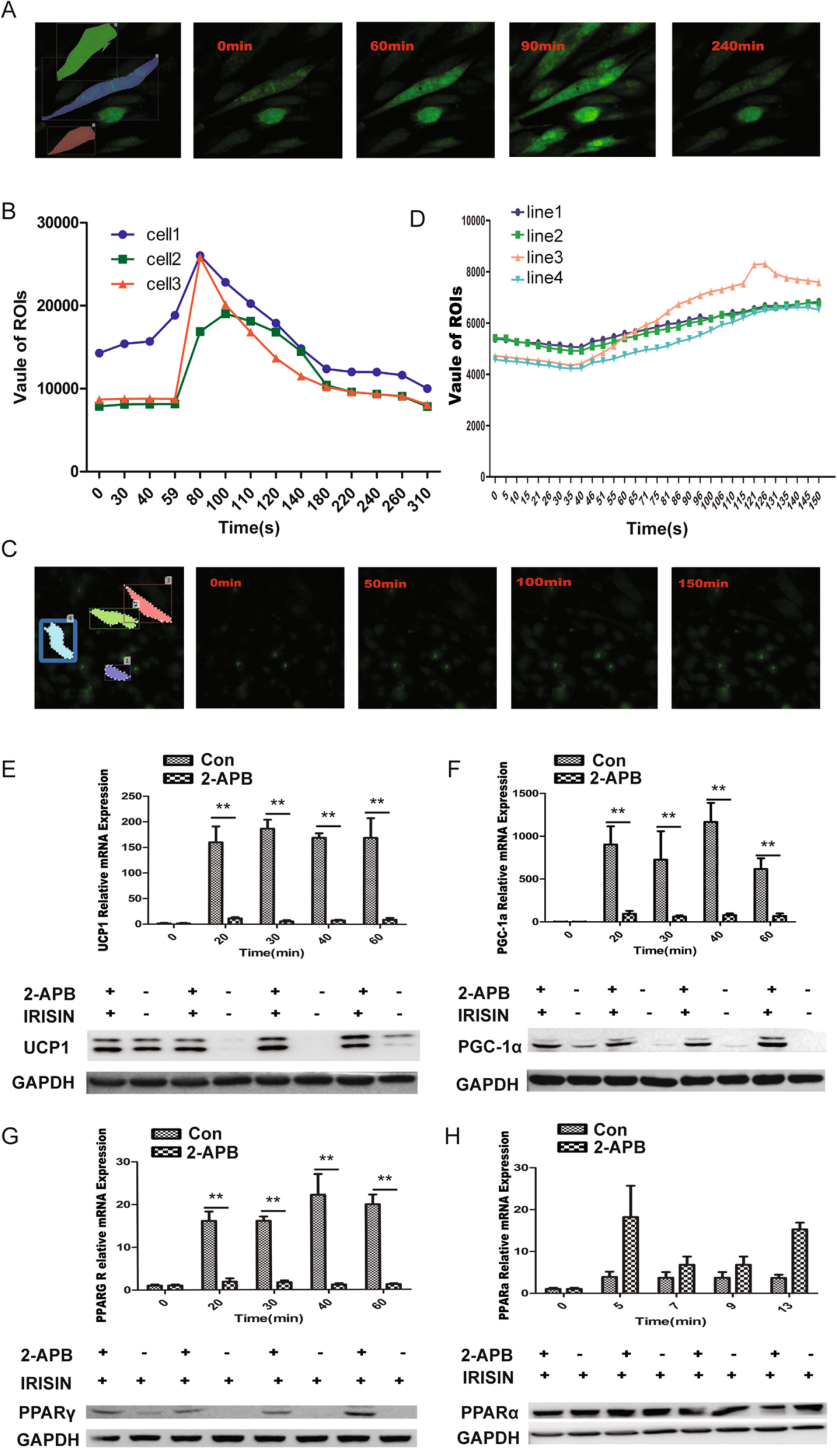
Treatment with 2-APB inhibits calcium influx and attenuates IRISIN-induced beige cell differentiation. **A** Fluo-4-stained live cells stimulated with IRISIN were observed under a two-photon microscope after 60 s. The change in intracellular calcium ion concentration was monitored in real time. The calcium ion concentration increased significantly. After reaching the highest point, the fluorescence intensity gradually decreased. **B** The real-time intracellular calcium concentration was measured by two-photon microscopy and expressed as the fluorescence intensity. **C** Two-photon microscopy was used to monitor the intracellular calcium concentration after treatment with 2 μM of 2-APB for 2 h and 4 μM Fluo-4 AM for 30 min. **D** Fluorescence intensity was measured by two-photon microscopy after treatment with 2-APB. There was no significant change in intracellular calcium before and after IRISIN stimulation. **E** *UCP1* gene expression was quantified by measuring real-time fluorescence intensity. UCP1 protein expression was evaluated by western blotting. UCP1 expression was significantly inhibited upon 2-APB treatment. **F** *PGC-1α* gene expression was quantified by measuring real-time fluorescence intensity. PGC-1α protein expression was determined by western blotting. PGC-1α expression was significantly inhibited upon 2-APB treatment. **G** *PPARγ* gene expression was quantified by measuring the real-time fluorescence intensity. PPARγ protein expression was detected by western blotting. PPARγ was significantly inhibited (*P* < 0.01) upon 2-APB treatment. **H** *PPARα* gene expression was quantified by measuring real-time fluorescence intensity. PPAR*α* protein expression was detected by western blotting. PPARα expression did not decrease but tended to increase upon 2-APB treatment.*p < 0.05, **p < 0.01, and ***p < 0.001

TRPC3 is a membrane protein that can form a non-selective channel permeable to calcium. We next examined whether TRPC3 was involved in regulating the Ca^2+^ influx in beiging adipose cells. IRISIN treatment in beiging adipose cells was found to significantly stimulate the Ca^2+^ influx using Fluo-4 staining (Fig. 3A, B). At the same time, we found the enhancement of electric current in the MSC cells by chip detection (Fig. S1). The mammalian TRPC subfamily contains seven structurally conserved members. It is reported that the structures of TRPC3 and TRPC6 are similar [40]. It is thus necessary to confirm whether other family members might be involved in IRISIN-induced Ca^2+^-related pathway activation. We analyzed the interaction between IRISIN and TRPC3/4/6 in 293T cells, which do not express these receptors in their natural state. Using TRPC3/4/6 ectopically expressed in 293T cells, we observed that the intracellular calcium ion concentration in TRPC3 overexpressing 293T cells significantly increased upon IRISIN stimulation (Fig. S2A). IRISIN could not induce calcium influx through TRPC4 (Fig. S2B), in TRPC6 overexpressing 293T cells, IRISIN also induced calcium ion influx (Fig. S2C), mainly because TRPC4 has a different structure compared to those of TRPC3 and TRPC6. MSCs expressed TRPC3 and TRPC4 but did not express TRPC6 protein (Fig. S2E). Phosphorylated calcium-related proteins PKA and ATP-2A were detectable by western blotting, and the levels of these proteins were markedly increased upon IRISIN stimulation (Fig. S3). Thus, TRPC3 represents newly identified factor in MSCs that play essential roles in the IRISIN-induced beiging of MSCs.

The TRPC3 antagonist 2-APB was used to test whether blocking the channels could inhibit IRISIN-induced beiging of MSCs. There was an obvious inhibition of the IRISIN-stimulated calcium influx in 2-APB treatment (Fig. 3C, D). 2-APB treatment also decreased the expression levels of thermogenic genes (*UCP1*, *PGC-1α*, and *PPARγ*) (*P* < 0.05) (Fig. 3E–G), whereas it had no effect on white fat-related *PPARα* gene expression (Fig. 3H). Consistently, small interfering RNA (siRNA)-mediated *TRPC3* knockdown decreased the expression of thermogenic genes *UCP1*, *PGC-1α*, and *PPARγ* compared to that in control siRNA-treated cells, whereas *PPARα* expression was not affected. Finally, the results of CRISPR-Cas-mediated *TRPC3* knockout are also consistent with those of 2-APB treatment and siRNA treatment (Fig. S4). These results suggest that *TRPC3* is essential for IRISIN-mediated beige differentiation of MSCs.

### IRISIN interacts with TRPC3 to activate the ERK/AKT signaling pathway

**Fig. 4:**
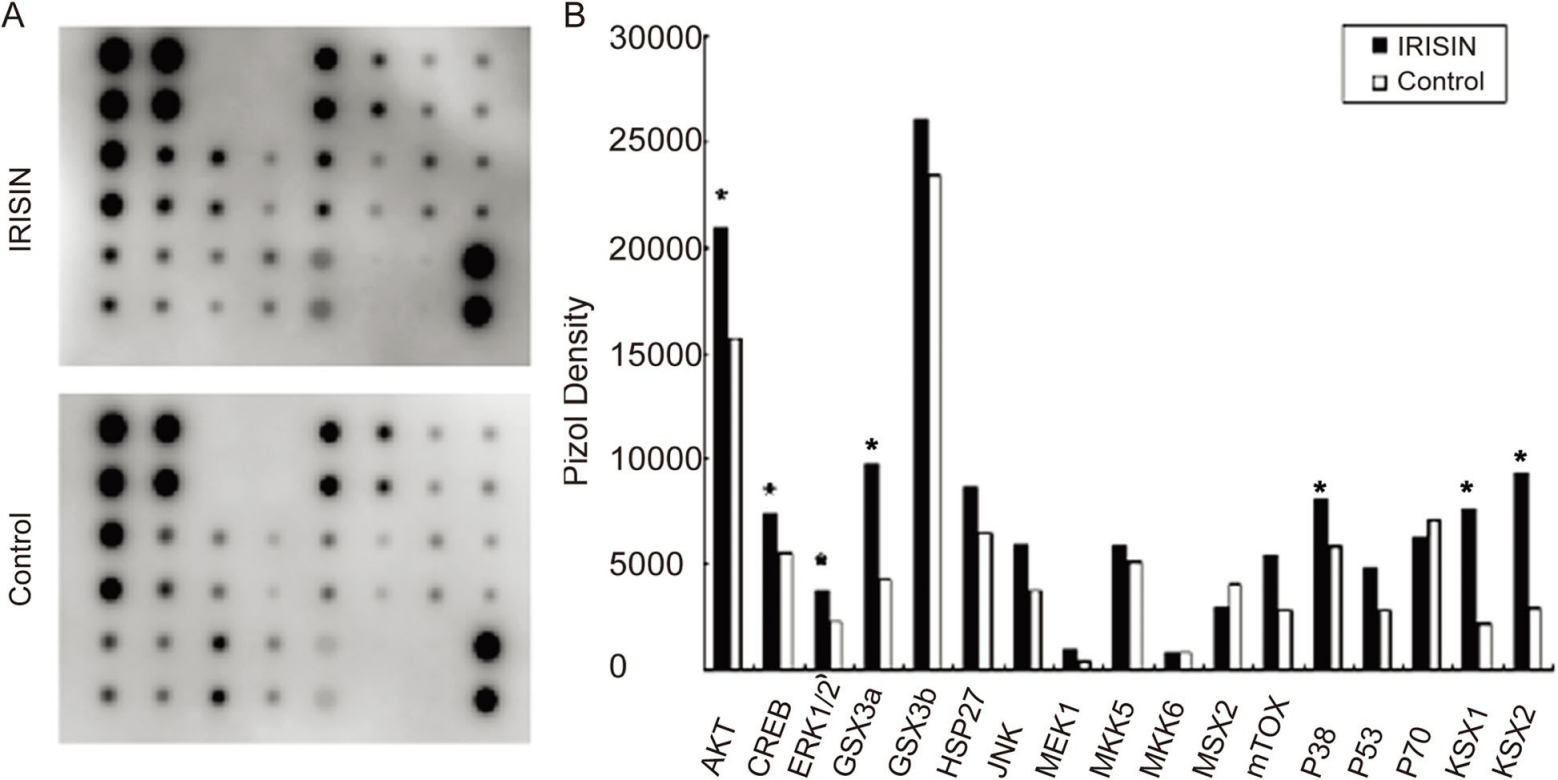
Analysis of the downstream signaling pathway after IRISIN stimulation. **A, B** Rosiglitazone induces differentiation of mesenchymal stem cells into beige fat cells. The cells are divided into two groups: the untreated group and the IRISIN-treated group. Chemiluminescence was analyzed using the AAH-MAPK-1 software. There was a significant change in the AKT, ERK, CREB, and P38MAPK, RSK1/2 signaling pathways. *p < 0.05, **p < 0.01, and ***p < 0.001

**Fig. 5:**
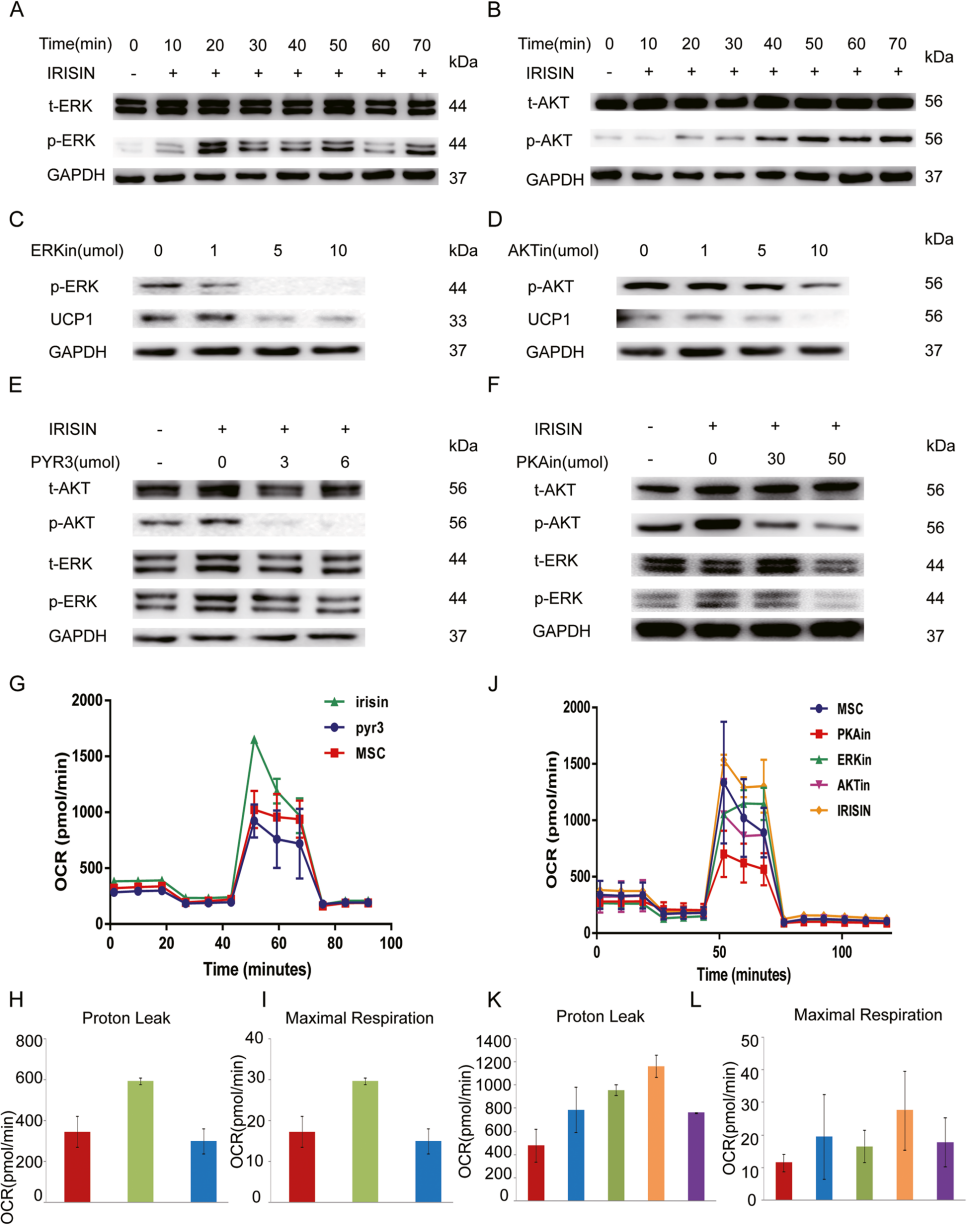
IRISIN stimulates browning of MSCs via AKT/ERK pathways. **A** Adipose-derived mesenchymal stem cells (AD-MSCs) were treated with IRISIN (200 nmol/L) at the indicated time points. The levels of phosphorylated and total ERK, ERK1/2 protein in the cell lysate were analyzed by western blotting in the IRISIN-induced beige cells. **B** Adipose-derived mesenchymal stem cells (AD-MSCs) were treated with IRISIN (200 nmol/L) at the indicated time points. The levels of phosphorylated and total AKT protein in the cell lysate were analyzed by western blotting in the IRISIN-induced beige cells. **C, D** AD-MSCs were pretreated with AKT inhibitor or ERK inhibitor (U0126) at the indicated concentrations for 12 h followed by IRISIN treatment. Phosphorylated AKT, ERK, and UCP1 were detected by western blotting. **E** AD-MSCs were pretreated with TRPC3 inhibitor(PYR3) at the indicated concentrations followed by IRISIN treatment. Phosphorylated and total AKT and ERK proteins were detected by western blotting. **F** AD-MSCs were pretreated for 4 h with PKA inhibitor at the indicated concentrations followed by IRISIN treatment. Phosphorylated and total AKT and ERK were detected by western blotting. **G** Beige cells were treated with TRPC3 inhibitors (PYR3, 6μM). After treatment for 12 h, the Seahorse XF Extracellular Flux Analyzers detected the mitochondrial respiration rate in beige fat cells. The respiration rate of the treated group was significantly lower when compared to that of the untreated group (*P* < 0.05). **H, I** The ability to detect proton leak and maximal respiratory capacity when the baseline was consistent with the presence or absence of IRISIN stimulation. **J** Beige cells were treated with PKA, ERK, and AKT inhibitors (2 μM). After treatment for 12 h, the Seahorse XF Extracellular Flux Analyzers detected the mitochondrial respiration rate in beige fat cells. The respiration rate of the treated group was significantly lower when compared to that of the untreated group (*P* < 0.05). **K, L** The ability to detect proton leak and maximal respiratory capacity when the baseline was consistent with the presence or absence of IRISIN stimulation

We used a protein chip with a calcium-related signaling pathway to initially screen the downstream signaling pathways involved in TRPC3 activation upon IRISIN stimulation. We analyzed these signaling pathways using the AAH-MAPK-1 software. We observed that AKT, ERK, CREB, P38MAPK, and RSK1/2 signaling pathways were altered upon IRISIN stimulation (Fig. 4A, B). Western blotting analysis showed that ERK phosphorylation was transiently induced after IRISIN stimulation (Fig. 5A). After IRISIN stimulation, AKT started to be phosphorylated and continued increasing (Fig. 5B). UCP1 expression was downregulated upon treatment with inhibitors of ERK and AKT signaling pathway (Fig. 5C/D). We next tested whether TRPC3 and its channel-associated protein PKA are involved in regulating the ERK and AKT activation pathway. We therefore inhibited TRPC3 and PKA in beige fat cells and found that this markedly impaired the activation of ERK and AKT pathways (Fig. 5E/F). These observations were further validated by the significantly reduced oxygen consumption rate in TRPC3-, PKA-, ERK-, and AKT-inhibitor-treated beige fat cells compared to the untreated group (*P* < 0.05) (Fig. 5G, J). The mitochondrial respiration rate reflected an increase in the proton leak and maximal mitochondrial respiration in cells, consistent with the same baseline levels as the untreated group (Fig. 5H, I, K, L). These results uncovered that TRPC3 is essential for IRISIN-mediated beige differentiation of MSCs.

### IRISIN promotes UCP1 expression by regulating PPARγ phosphorylation

**Fig. 6:**
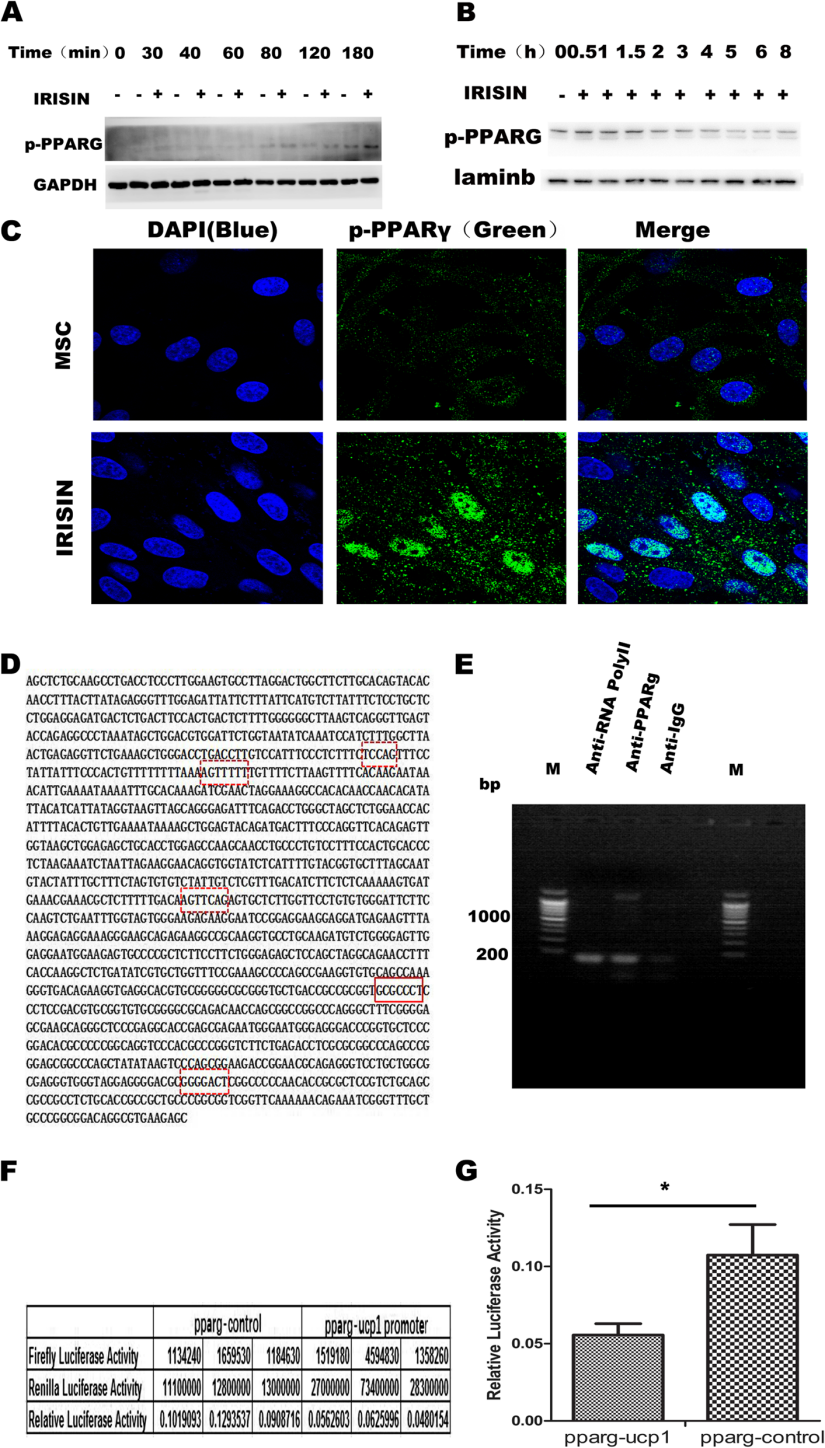
PPARγ promotes beige cell differentiation of MSCs by binding the *UCP1* promoter. **A** After IRISIN stimulation for different durations, p-PPARγ expression in cells was detected by western blotting. **B** After IRISIN stimulation for different durations, the cell lysate was prepared by nucleolysis. The expression of p-PPARγ in the nucleus was detected by western blotting. **C** After IRISIN stimulation for 7 days, PPARγ phosphorylation was detected in the nucleus using a microscope. Compared to the control group, the level of phosphorylated PPARγ was higher in the nucleus of the IRISIN-treated group. **D, E** In the presence or absence of IRISIN stimulation, the two groups of cells were fixed with formaldehyde, and the fragment was sonicated; the PPARγ antibody captured the binding fragment and PCR was used to detect the binding fragment. **F, G** The *UCP1* promoter was introduced into the PGL4.10 vector (PGL4.10-*UCP1* promoter). PGL4.74 was used as the internal reference vector. PGL4.10-*UCP1* promoter and PPARγ overexpression vectors were co-transfected into 293T cells. The control cells were co-transfected with PGL4.74 and *PPARγ* vectors. *PPARγ* overexpression was detected by the dual luciferase assay. The experiment was repeated three times. The results of dual luciferase were analyzed using primer5. The results revealed significant interaction between PPARγ and the *UCP1* promoter (*P* < 0.05). *p < 0.05, **p < 0.01, and ***p < 0.001

We observed that PPARγ expression was significantly enhanced upon IRISIN stimulation as mentioned above. The phosphorylated PPARγ (p-PPARγ) in total protein and nuclear protein began to increase 2 h after IRISIN stimulation (Fig. 6A, B). Immunofluorescence staining also revealed that PPARγ phosphorylation in the nucleus was significantly increased with IRISIN stimulation (Fig. 6C). To examine whether S112 phosphorylation of PPARγ affected the beiging of MSCs, we performed chromatin immunoprecipitation followed by PCR and dual luciferase assay. ChIP assay revealed that p-PPARγ could bind the promoter region GCGCCCT, at − 384bp to − 392bp upstream in the UCP1 promoter (Fig. 6D/E). Using a luciferase reporter coupled with *UCP1*-responsive promoter sequences, we confirmed that PPARγ overexpression increased *UCP1*-dependent transcription significantly (Fig. 6F/G). These results suggested that nuclear phosphorylated PPARγ could promote the beige differentiation of MSCs by regulating UCP1 expression.

**Fig. 7:**
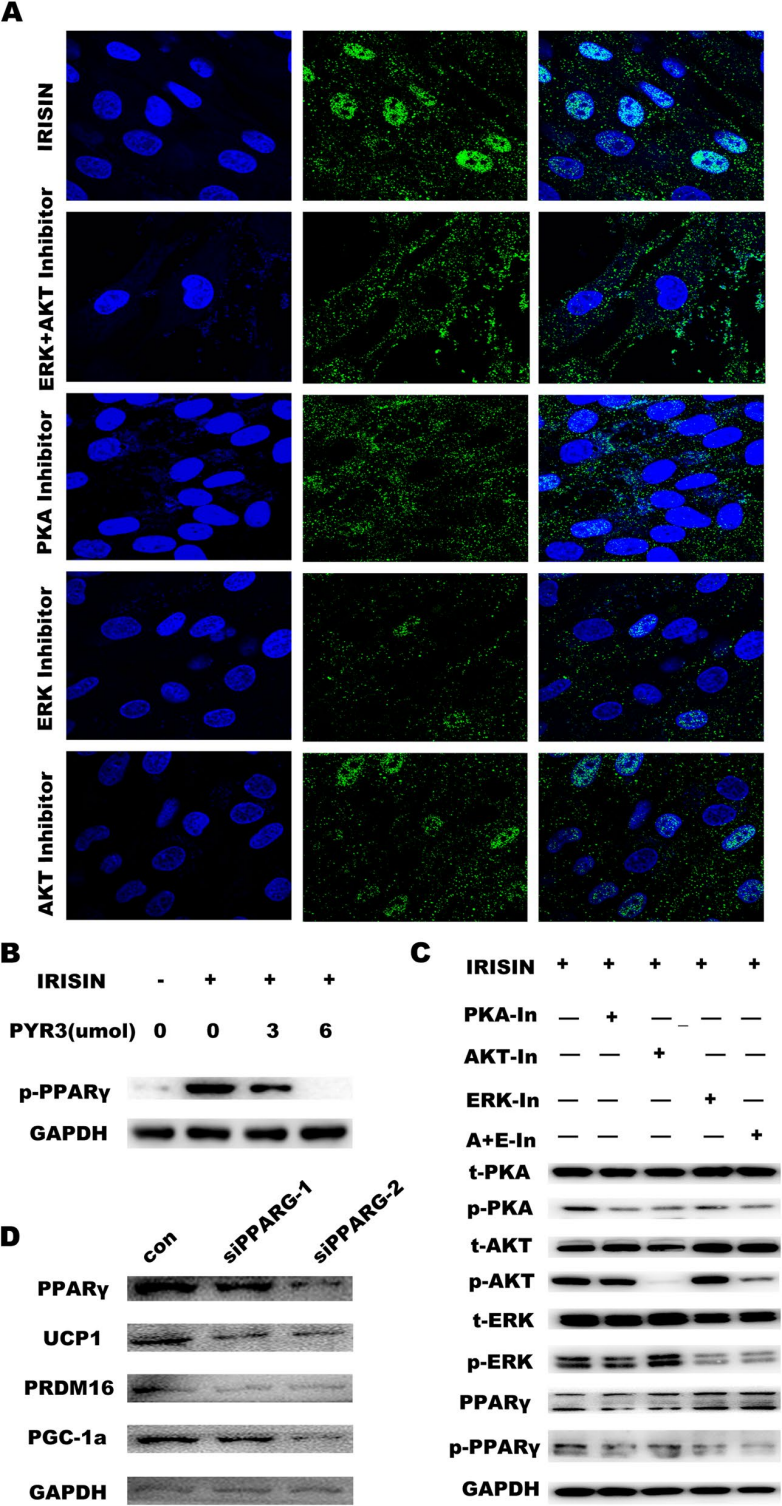
ERK/AKT signaling pathway promotes the entry of phosphorylated PPARγ into the nucleus. **A** Upon IRISIN stimulation, treatment with the inhibitors of PKA, ERK, AKT, and co-treatment with the inhibitors of ERK and AKT, phosphorylation of PPARγ was observed by confocal microscopy. **B** Phosphorylation of the proteins was detected by western blotting. Expression of phosphorylated PPARγ protein with PYR3 inhibitor. **C** Phosphorylation of the proteins was detected by western blotting. Expression of phosphorylated PPARγ protein with the inhibitors of PKA, ERK, AKT, and co-treatment with the inhibitors of ERK and AKT. **D** Western blotting was used to detect the expression of beige fat marker genes UCP1, PGC-1α, and PRDM16

We then checked the role of TRPC3 in modulating PPARγ phosphorylation by immunofluorescence staining. An inhibitor of PKA significantly decreased the phosphorylation levels of PPARγ in cultured beiging cells. Upon treatment with inhibitors of the ERK or AKT signaling pathway, PPARγ phosphorylation was partially inhibited. However, upon co-treatment with ERK and AKT signaling pathway inhibitors, PPARγ phosphorylation was completely inhibited (Fig. 7A). The western blot results were consistent with these results, phosphorylated PPARγ protein levels were decreased upon treatment with the TRPC3 /PKA/ERK/AKT inhibitors and co-treatment with ERK and AKT inhibitors (Fig. 7B/C). UCP1, PGC-1α, and PRDM16 expressions were significantly downregulated upon PPARγ knockdown (Fig. 7D). These results reveal that IRISIN binding to TRPC3 protein leads to calcium influx, PKA phosphorylation, and ERK and AKT pathway activation, which finally phosphorylates PPARγ and mediates its nuclear entry. Phosphorylated PPARγ in the nucleus binds to the promoter region of UCP1 and promotes UCP1 expression, which enhances the beige differentiation of MSCs.

### Trpc3 mediates IRISIN-induced thermogenesis in vivo

**Fig. 8:**
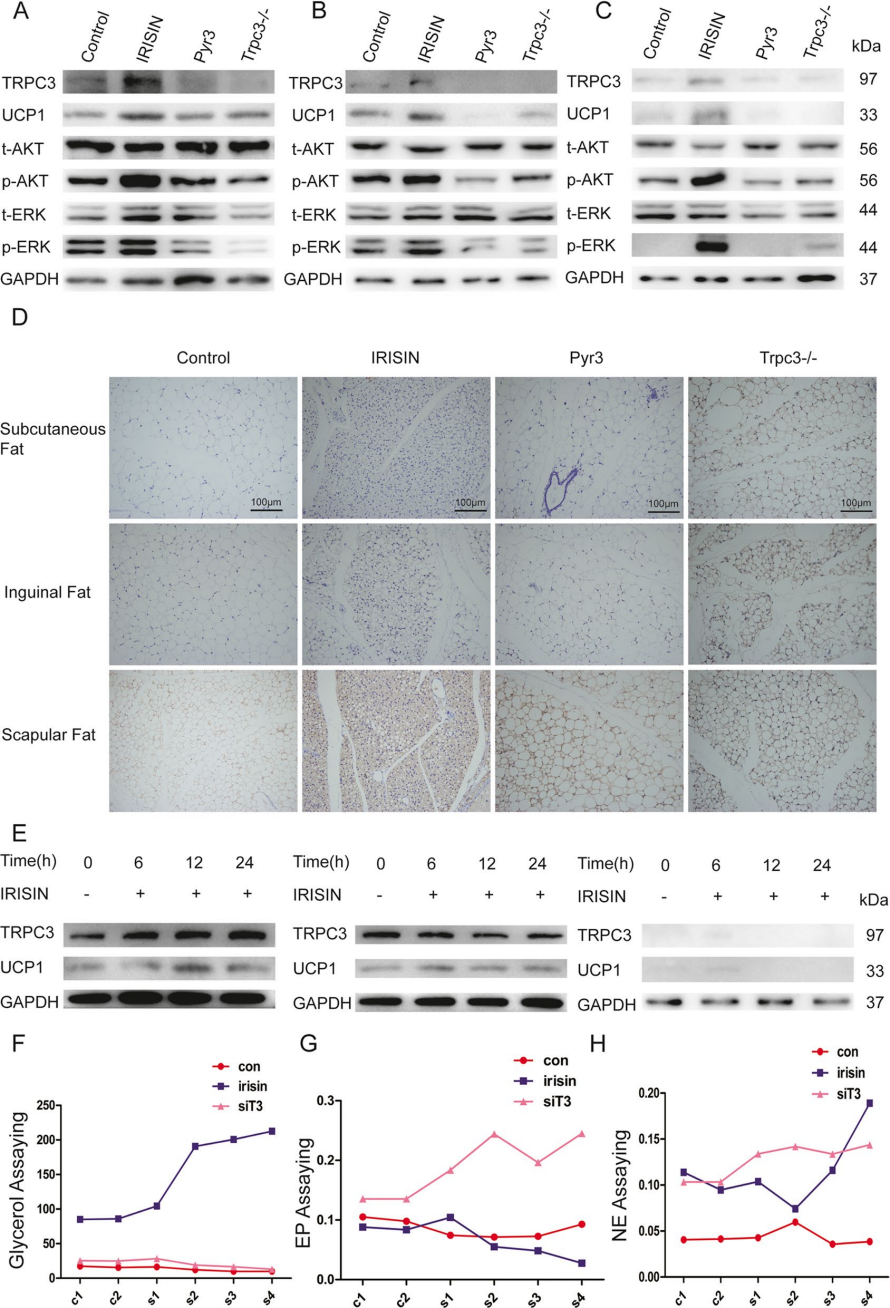
*Trpc3* mediates IRISIN-induced thermogenesis in vivo. **A-C** Different parts of adipose tissues obtained from a normal C57BL/c wild group, IRISIN-treated C57BL/c wild group, combination with IRISIN and TRPC3 inhibitor (PYR3)-treated C57BL/c wild group, and IRISIN-treated *Trpc3*^−*/*−^ knockout C57BL/c group were lysed to detect the protein levels of UCP1, p-ERK, and p-AKT by western blot. **D** Adipose tissues obtained from different parts were lysed to detect the protein levels of UCP1 using immunohistochemistry. **E** Mesenchymal stem cells (MSCs) were isolated from the adipose tissue of wild-type mice, *Trpc3* heterozygous mice, and *Trpc3*^−*/*−^ knockout mice. IRISIN was used to stimulate these MSCs. Cells were collected from the three groups of mice at 0, 6, 12, and 24 h after stimulation. Western blotting was used to detect the expression of UCP1 proteins. **F–H** Microdialysis method was used to detect the metabolites such as glycerin, epinephrine, and norepinephrine in adipose dialysate of C57BL/c wild group, IRISIN-treated C57BL/c wild group and IRISIN-treated *Trpc3*^−*/*−^ knockout C57BL/c group

To further test the function of IRISIN in the beiging of white adipose tissue in vivo, we established animal models to verify the effects. Four groups were set: normal C57BL/6J wild group, IRISIN-treated C57BL/6J wild group, IRISIN and TRPC3 inhibitor (PYR3)-treated C57BL/6J wild group, and IRISIN-treated *Trpc3*^−*/*−^ C57BL/6J group. On day 15 after IRISIN treatment, we found that the IRISIN and PYR3 combination-treated group and IRISIN-treated *Trpc3*^−*/*−^ C57BL/6J group showed obviously lower expression of UCP1 and phosphorylated ERK and AKT, compared to the IRISIN-treated C57BL/6J wild group in the subendothelial fat, inguinal, and scapular adipose tissue (Fig. 8A–D, Fig. S5). Simultaneously, adipose-derived MSCs from C57BL/6J wild mice, *Trpc3*^−*/*−^ heterozygous mice, and *Trpc3*^−*/*−^ mice were isolated and stimulated with IRISIN in vitro. After 6, 12, and 24 h, UCP1 expression was not detected in adipose-derived MSCs from *Trpc3*^−*/*−^ mice (Fig. 8E). In addition, we detected the metabolism of glycerol, epinephrine and norepinephrine in the fatty dialysate of wild mice, IRISIN-treated mice, and IRISIN-treated *Trpc3*^−*/*−^ mice by microdialysis. The results showed that compared with the other two groups, the changes of norepinephrine and glycerol in IRISIN-treated mice were significant, but the changes of epinephrine were not significant (Fig. 8F–H). These results indicate that TRPC3 is necessary for IRISIN-induced MSC differentiation into beige cells and mediates energy metabolism.

## Discussion

Since the concept of MSCs has been proposed, which has been reported to play a positive therapeutic role in the treatment of various diseases, these functions are mainly related to the differentiation potential of MSCs and do not form teratoma [41–43]. It has been studied that all fat cells originate from MSCs, so it is important to understand whether the cell differentiation direction is beneficial to humans [30]. Moreover, the mechanisms controlling these differentiation directions are not clear. IRISIN acts as a hormone produced after exercise, and its function is similar to the effects of exercise such as increasing energy expenditure and improving cognition and cortical bone [23, 44]. IRISIN is known to promote the conversion of white adipocytes into beiging adipocytes [22, 24], but its regulation in MSC has not been found. IRISIN can be produced after exercise, which can directly regulate the differentiation of MSCs into the beneficial beige fat cells, further explaining that exercise can lead to weight loss. In our research, first, we verified that MSCs could differentiate into beige fat cells by detecting the beige fat marker genes. Second, MSCs derived beige fat cells showed ATP energy consumption in form of heat, which is beige fat cells’ important character in energy metabolism. Finally, we monitored that the mitochondrial aerobic respiration was significantly enhanced, which is brown fat cells’ respiration pattern. Together, these results suggested that IRISIN can enhance the differentiation of MSCs into functional beige fat.

Studies in cell culture systems have implicated TRPCs in a wide variety of physiological processes in mammals ranging from muscle relaxation/contraction, fluid secretion, growth cone guidance, and morphology, to acrosome reaction [45–47]. In addition, phospholipase Cgamma1 (PLC-gamma1) binds to and regulates TRPC3 channels, components of agonist-induced Ca^2+^ entry into cells [48]. All TRPC channels are also activated by receptor stimulation, and several TRPCs have been suggested to function as SOCs [49–51]. And several studies showed that TRPC3 behaves as a store-operated Ca^2+^ channel (SOC) [49, 52, 53]. Our study first explains that MSC can respond to IRISIN through their membrane surface receptor TRPC3. We performed IRISIN IP analysis with MSC cell lysates, followed by MS analysis with the proteins pulled down from IRISIN. TRPC3 was screened as an IRISIN interacting protein candidate with western blot confirmation. Subsequently, we used biofilm interference (BLI) experiments to detect interaction of IRISIN and TRPC3. In addition, function and loss-function of TRPC3 assays dedicated that the extracellular calcium influx and the cell membrane potential changes in MSC cells after IRISIN stimulation, which is consistent with the calcium channel characteristics of TRPC3. Existing reports indicate that IRISIN not only induced mitochondrial biogenesis with the subsequent upregulation of mitochondrial genes (TFAM, NRF1, NRF2 or UCP1) but regulated energy expenditure of adipocyte [54–57]. In our experiments, we found that TRPC3 was clearly activated after IRISIN was added to MSC cells. After knocking down the TRPC3 gene, the ability of MSCs to differentiate into beige fat cells was significantly attenuated by inhibiting extracellular calcium influx. Moreover, inhibition of TRPC3 significantly attenuated mitochondrial respiration of beige fat cells. Our results also demonstrated that IRISIN could directly activate TRPC3, but it has no obvious effect on its family of TRPC4/6. In addition, IRISIN-induced beiging was inhibited in *Trpc3*^−*/*−^ mice, revealing a potential treatment for regulation of energy metabolism through targeting IRISIN and TRPC3. Based on these ex vitro and in vivo data, we concluded that TRPC3 plays a key role in regulating IRISIN to promote MSC differentiation into beige fat. Recently, a subset of integrins, especially those involved aV integrin was reported as the functional receptors of IRISIN in osteoocytes and fat tissues in 2018 [29]. But whether this pathway works in our system remains to be tested in the future.

Several studies have revealed that IRISIN can stimulate intracellular calcium signaling pathways. For example, IRISIN can control growth and mitochondrial heat production through Ca^2+^ signals in cardiomyocytes [28]. In endothelial cells, Irisin-induced diastemia-dependent vasodilation is related to the stimulation of extracellular Ca^2+^ influx via TRPV4 channels [43]. Existing reports indicate that IRISIN stimulates expression of UCP1 in white adipocytes which lead to browning via p38 mitogen-activated protein kinase (MAPK) and extracellular-signal regulated kinase (ERK) pathways [22, 25], and FNDC5/IRISIN improves insulin resistance and glucose/lipid metabolic derangements and strengthen lipolysis via cAMP-PKA-HS-L/perilipin pathway in obese mice [58]. However, no studies have shown how IRISIN activates intracellular calcium signaling pathways. In addition, the role of IRISIN in beige fat cells through TRPC3 has not been reported. In our study, we used a protein chip with a calcium-related signaling pathway to initially screen the downstream signaling pathways. Screening results showed that IRISIN binding with TRPC3 proteins induced PKA phosphorylation which activates the downstream ERK and AKT signaling pathways. Therefore, IRISIN may exert biological functions on different tissues through different receptors and downstream diverse signaling pathways.

Although the receptor for IRISIN has been identified in bone and adipose tissue [29], TRPC3 is not excluded as another functional receptor for IRISIN. In this paper, we did not resolve the protein structure of TRPC3 and IRISIN interaction and hope to further solve the binding characteristics between IRISIN and TRPC3 proteins which can lay a solid foundation for the analysis of the unknown function of IRISIN. Importantly, the identification of IRISIN interacting proteins and their associated signaling pathways is of great interest for the treatment of IRISIN in future metabolic diseases and its functional studies. In addition, this discovery also helped us to further understand the TRPC calcium-related protein family, which laid the foundation for its subsequent research. Finally, our future research should focus on tissues with high expression of TRPC3 (brain), and study whether IRISIN positively regulates cognition by interacting with TRPC3 and activating related signaling pathways. These functions should be obtained for further verification in subsequent studies.

## Conclusions

The myogenic factor, IRISIN, is important for cellular energy metabolism. Our data demonstrate that IRISIN can promote the differentiation of MSCs into beige fat cells. The ability of IRISIN to induce the differentiation of MSCs into beige cells was higher than that of rosiglitazone and other drugs. We demonstrate that IRISIN binds to TRPC3 and promotes calcium influx, which activates PKA-related proteins. The calcium influx also promotes PKA phosphorylation, which activates the downstream ERK and AKT signaling pathways. The phosphorylated ERK and AKT phosphorylates PPARγ protein, which enters the nucleus and promotes UCP1 expression, a beige fat cell marker gene, by binding the *UCP1* promoter. UCP1 induces the differentiation of MSCs into beige cells. Hence, IRISIN could be used to develop an in vitro model for beige fat cell differentiation of MSCs, laying the foundation for later metabolic diseases. The molecular mechanism study underlying beiging provide potential therapeutic targets for metabolic diseases.

## Methods

### Animals and protein preparation

All animal care, usage, and experiments were reviewed and approved by the Animal Use Committee of Animal Use Center of Peking Union Medical College.

*Trpc3* ^−*/*−^ mouse was derived from a mixed 129SvEv:C57BL/6J background, which was obtained from Dr Sun JP at Shandong University School of Medicine and also was maintained and crossed as described previously [59]. Male mice aged 6–8 weeks were screened for later experiments. TRPC3 protein was obtained from Chen Lei lab in Peking University [40].

### Isolation and culture of human AD-MSCs

Abdominal fat tissues were removed under aseptic conditions after obtaining informed consent from donors and were diluted with phosphate buffered saline (PBS) solution. The sample was centrifuged at 800*g* for 3 min. The clear bottom solution was discarded using a pipette. Collagenase was added to the liquid fat at one fourth the volume of fat and incubated on a horizontal shaker at 37 °C, and 200 rpm for 15–30 min. The digested adipose tissue was filtered through a 100-μm cell strainer with the appropriate amount of D-Hanks’ solution. Undigested tissue was removed, and the sample was centrifuged at 1500*g* for 10 min. The samples were washed twice with D-Hanks’ solution to remove the cells. The cells were collected by centrifugation. MSCs were cultured in complete MSC medium (10 mL) in a 10-cm dish. When the cells reached 80–90% confluency, the cells were digested and subcultured to obtain the third generation (P3), which was used after identification.

### Adipogenic differentiation and beige cell differentiation of AD-MSCs

The white adipogenic differentiation induction medium was prepared by adding IBMX (final concentration 0.5 mM), dexamethasone (final concentration 1 μM), and ascorbic acid (final concentration 0.1 mM) to DMEM, high-glucose (H-DMEM) supplemented with 10% fetal calf serum. The human AD-MSCs were cultured till they reached 90% confluency. Then, the MSC culture medium was replaced with white/beige adipogenic differentiation induction medium. The induction medium was changed once every 2 days. The formation of lipid droplets in cells was observed under an inverted microscope. The cells were subjected to Oil red O staining for about 15 min and were then observed under a microscope (Olympus).

### Establishment of the in vitro beiging fat model

Beiging fat differentiation induction medium was prepared by adding ascorbic acid (final concentration 0.1 mmol/L), rosiglitazone (final concentration 200 nmol/L), and IRISIN (final concentration 100 nmol/L) to DMEM/F12 medium supplemented with 10% fetal bovine serum. Human AD-MSCs were grown until they attained a confluency of 90%. Next, the MSC medium was replaced with the beiging fat differentiation induction medium. The induction medium was replaced every 2 days. The matured cells were then used for further analysis.

### Detection of cellular immunofluorescence

Approximately 2 × 10^5^ cells were seeded in a 6-well plate and induced to maturity using the beiging induction medium. The cells were then fixed using 4% paraformaldehyde and permeabilized using 0.3% Triton X. The permeabilized cells were washed twice with PBS and probed with UCP1 antibody (1:100, Peprotech) for 1 h. Cells were then washed twice with PBS on a horizontal shaker at room temperature (24–26 °C) and 40 rpm for 5 min. The cells were then incubated with the FITC-goat anti-rabbit IgG secondary antibody (Millipore) for 30 min. Cells were washed twice with PBS, and staining patterns were examined using an FV1200 confocal microscope (Olympus).

### Real-time polymerase chain reaction (RT-PCR)

RNA from cell samples was extracted using Trizol (Thermo Fisher Scientific) following the manufacturer’s instructions. RNA was eluted in 30 μL RNA-free water (Applygen), aliquoted, reverse transcribed (60 μL), and treated according to the protocol recommended for the TaKaRa M-MLV reverse transcriptase (Takara). Amplification of the gene fragment was performed as follows: 72 °C for 60 min, 42 °C for 10 min. Real-time PCR experiments were performed following the manufacturer’s procedure for the Takara SYBR® Premix Ex TaqTM kit. To amplify the *UCP1*, *PPARγ*, *PGC-1a*, *TRPC3*, *PPARa*, and *GAPDH* genes, one-step RT-PCR was performed. Amplification of gene fragments was performed as follows: 95 °C for 5 min, 95 °C for 10 s, 60 °C for 40 s, 40 cycles. Reactions were performed in triplicate, and independent experiments were repeated three times. The RT-PCR data were analyzed using StepOne Software 2.1.

### Western blotting

We detected the protein bands on the membrane by enhanced chemiluminescence using Millipore ECL kit, following the manufacturer’s instructions. Briefly, cells were harvested in RIPA lysis buffer (Beyotime) with 1 mmol phenylmetha- nesulfonyl fluoride; lysates were quantified using a BCA Protein Assay Kit (Beyotime), separated by 10% SDS-PAGE, and transferred to polyvinylidene fluoride membranes (Millipore). The membranes were blocked with 5% non-fat milk in TBST for 1 h and incubated with primary antibodies overnight at 4 °C. The primary antibodies were as follows: ERK(#4695, CST), p-ERK(Thr202/Tyr204, #4370, CST), AKT(#9272, CST), p-AKT(Ser- 473,#4060, CST), PPARγ(#95128, CST), p-PPARγ(PA5-36763, Thermo), UCP1(#97000, CST), PKA(#4782, CST), p-PKA (Ser/Thr, CST), TRPC3 (sc-514670, Santa Cruz), TRPC4 (21349-1-AP, Peprotech), TRPC6 (18236-1-AP, Peprotech), and then in horseradish peroxidase (HRP)-conjugated secondary antibodies for 1 h at room temperature. Results were observed with an Immobilon western chemiluminescent horseradish peroxidase (HRP) substrate (Millipore) and detected using a Tanon 4800 imaging system (Tanon).

### Mito stress test

The rates of oxygen consumption in beiging cell lines were measured using a Seahorse Bioscience XF-96 Extracellular Flux Analyzer (Seahorse Bioscience). An example protocol is listed below for beiging cell lines. Thermophilic DMEM and trypsin were incubated in a 37 °C water bath. Cells were trypsinized and suspended in the complete medium. The cell suspension was centrifuged for 3 min at 600*g* to precipitate cells. The culture medium/trypsin supernatant was discarded, and the cells were suspended in 5 mL complete culture medium. Cells were counted on a blood cell counter, and the number of living cells per milliliter of the medium was determined. The amount of seeding required is variable; however, in our experience, the cell number should be between 10,000 and 80,000 cells, depending on the cell size and respiratory rate. Wells A1, B4, C3, and D6 were kept empty to monitor the temperature fluctuations in the plates and to correct background. Sterile filtration medium was transferred (50 mL) to a clean 50-mL conical tube. The medium of the equal samples was heated to 37 °C in a water bath. The pH of the medium was adjusted to 7.4 with 0.1N NaOH. The medium was removed from the cell plate, and 50 μL was left in the pore. This was accomplished by careful suction or by the use of a multichannel pipette. The multichannel pipette was used to wash cells with 1 mL thermometric medium. The determination medium was removed from the pore, and 50 μL was left in the pore again. A multichannel pipette was used to add 625 μL of medium to each pore to a final volume of 675 μL. The plate was placed in a non-CO_2_ incubator at 37 °C for 1 h. The final concentration of 3 mL oligomycin was 10 μg/mL. This concentration was 10 times the desired final concentration because once injected into the measurement hole, the solution would be diluted 10 times. This concentration was suitable for cells. The final concentration of 3 mL FCCP was 10 μmol/L. This concentration was the final concentration required for 10×. Then 3 mL of antimycin A was prepared in the determined medium to a final concentration of 100 mmol/L. This concentration was 10 times the required final concentration because once injected into the measurement hole, the solution would be diluted 10 times. Then 75 μL oligomycin solution was added to port A on the XF Assay column and 83.3 μL FCCP solution was added to port B on XF Assay column. Then, 92.6 μL antimycin A solution was added to port C on the XF Assay column. The XF Assay column was loaded into XF24 at 30 min after replacing the medium on the cell culture plate. After completion of calibration, the practical plate of the cell culture plate was replaced, and the measurement procedure was continued. The typical mixing/waiting/measuring cycle was 2 min/2 min/3 min. The board and box from XF24 were removed at the end of the experiment.

### Ca2+ dynamic monitoring

#### Preparation of Fluo-4 AM mother liquor

An appropriate amount of DMSO was added to Fluo-4 AM to prepare a stock solution of 1–5 mmol/L. The amount of DMSO to be added was calculated according to the molecular weight of Fluo-4 AM (MW 1096.95). For example, to prepare a stock solution of 1 mmol/L mother liquor, 45.6 μL DMSO was added to 50 μg Fluo-4 AM.

#### Preparation of Fluo-4 AM working solution

The stock solution was diluted using HBSS to obtain the working solution of different concentrations (1–5 μmol/L). We prepared 4 μM working solution by adding 4 μL of the stock (1 mmol/L) mother liquor to 1 mL HBSS. If we did not detect sufficient fluorescence of Fluo-4, 20% Pluronic F127 solution was added to the Fluo-4 AM/DMSO solution to a final concentration of 0.04–0.05%. Pluronic F127 prevents Fluo-4 AM from polymerizing in HBSS and aids its cellular entry. The culture medium was removed from pre-cultured cells, and cells were washed thrice with HBSS. The working solution of Fluo-4 AM was added to the cells and incubated at 37 °C for 10–60 min. The Fluo 4-AM solution was removed, and the cells were washed three times with HBSS to completely remove the residual Fluo 4-AM solution. Next, HBSS was added to the cells, and cells were incubated for approximately 20–30 min at 37 °C to ensure complete de-esterification of the AM moiety in the cells. Fluorescence was detected by laser confocal or fluorescence microscopy with an excitation wavelength of 494 nm and an emission wavelength of 516 nm.

### Protein mass spectrometry

#### Reagents and instruments

HPLC-grade acetonitrile (ACN) and formic acid, trifluoroacetic acid, ammonium bicarbonate, iodoacetamide, and dithiothreitol were purchased from Sigma (St. Louis, MO, USA). Sequencing grade trypsin was purchased from Promega (Madison, WI, USA). TripleTOF 5600 mass spectrometer from AB Sciex and an HPLC system from Waters (Milford, MA, USA) were used.

#### Protein digestion

The proteins were separated by SDS-PAGE. The gel slices examined and wash with H_2_O and 50 mM NH4HCO3. After dehydration with ACN, then reduced with 20 mM dithiothreitiol at 95 °C for 5 min and were alkylated with 55 mM iodoacetamide at room temperature in the dark for 45 min. Gels were washed with 50 mM NH4HCO3 for twice and dehydration with ACN. Then, the dried gels were resolved in 25 mM NH4HCO3 containing trypsin with weigh ratio of 1:50 (rypsin: protein) at 37 °C overnight. The digested peptides were collected and desalted with Ziptip C18. The purified peptide samples were delivered to LC-MS/MS.

#### LC-MS/MS analysis

Each sample was analyzed with a reverse-phase-C18 self-packed capillary LC column (75 μm × 100 mm). The eluted gradient was 5–30 % buffer B (0.1 % formic acid, 98 % ACN; flow rate with 0.3 μL/min) in 30 min total LC duration. A TripleTOF 5600 mass spectrometer was used to analyze eluted peptides from LC. The MS data were acquired using high-sensitivity mode with following parameters:

Source gas1 is 20 and curtain gas is 20. Voltage is 2300 V. Scan type is positive. Mass range m/z is 350–1250. Thirty data-dependent MS/MS scans followed full scan acquired at high resolution, rolling collision energy, and charge state screening (including precursors with +2 to +4 charge state).

#### Data processing

For database searching, all MS/MS samples were analyzed using Protein Pilot (AB Sciex, version 5.0.1). Mascot was set up to search the SwissProt human database (20,227 entries) assuming the digestion enzyme Trypsin. The parent and fragment ion mass tolerance were 0.05 Da, respectively. Carbamidomethyl of cysteine was specified as a fixed modification, and 2 mis-cleavage sites were allowed. Protein identification was accepted at false discovery rate (FDR) less than 1.0% on a protein level.

### Immunoprecipitation/coprecipitation

The 293T cells were transfected with a *TRPC3* overexpression plasmid (NM_003305.2, GeneCopoeia). The transfected cells were treated with IRISIN protein 100 ng/μl (purity >95%, Sigma). The proteins were isolated from the cells and subjected to immunoprecipitation using Pierce Co-Immunoprecipitation (Co-IP) Kit (88828, Sigma, USA). The culture medium was carefully removed from cells, and the cells were washed with 1× modified Dulbecco’s PBS. Next, ice-cold wash buffer/IP lysis buffer was added to the cells, and the cells were incubated on ice for 5 min with mixing. The cell lysate was transferred to a microcentrifuge tube and centrifuged at ~13,000*g* for 10 min to discard the cell debris. The supernatant was transferred to a new tube for further analysis. The bottle of magnetic beads was vortexed to obtain a homogeneous suspension. Next, 25 μL of beads were added to a 1.5-mL microcentrifuge tube and the tube was placed on a magnetic stand for 60 s before collecting the beads. The solution was discarded and 400 μL ice-cold 1 mmol/L HCl was added to the tube, and gently vortexed to mix for 5 s. A magnetic stand was used to collect the beads and the supernatant was discarded. Next, 100 μL TRPC3 antibody solution was added to the beads, gently mixed, and incubated on a rotating platform for 30–60 min at room temperature (24–26 °C); during incubation, the beads were vortexed every 10–15 min to ensure that the beads remain in suspension. The beads were then collected with a magnetic stand. The flow-through was removed and saved for analysis. Next, 100 μL elution buffer was added and gently vortexed. The beads were collected using a magnetic stand, and the supernatant was discarded. This process was repeated once. The antibody solution was prepared using 10 μL borate buffer (0.67 mol/L) for diluting antibody stock and adding ultrapure water to the final volume 100 μL. The final concentration of the antibody was 5 μg/100 μL. Next, 500 μL of Quenching Buffer was added to the beads, gently mixed, and incubated on a rotating platform for 30–60 min. After discarding the supernatant, the beads were collected using a magnetic stand. Then, 0.5 mL of modified borate buffer for each IP reaction was prepared by diluting 25 μL of IP lysis/wash buffer with 475 μL of borate buffer. Then, 500 μL of modified borate buffer was added to the tube and gently vortexed or inverted to mix. The beads were collected on a magnetic stand and the supernatant was discarded. Then, 500 μL of IP lysis/wash buffer was added and gently vortexed or inverted to mix. The beads were collected on a magnetic stand, and the supernatant was discarded. The lysate solution was diluted with IP lysis/wash buffer to 1–2 mg/mL. Then, 500 μL of the diluted lysate solution was added to the tube containing the antibody-coupled magnetic beads and incubated for 2 h at room temperature on a rotator or mixer. The beads were gently vortexed every 15–30 min during incubation to ensure that the beads remained in suspension. The beads were then collected on a magnetic stand. The unbound sample was removed and saved for analysis. Next, 500 μL of IP lysis/wash buffer was added to the tube and gently vortexed or inverted to mix. The beads were collected on a magnetic stand, and the supernatant was discarded. This step was repeated once. Then, 500 μL of ultrapure water was added to the tube and gently vortexed or inverted to mix. The beads were collected on a magnetic stand and the supernatant was discarded. Then, 100 μL of elution buffer was added to the tube and incubated for 5 min at room temperature on a rotator or mixer, and the beads were magnetically separated. The supernatant containing the target antigen was retained. Then, 10 μL of neutralization buffer was added for each 100 μL of eluate to neutralize the low pH. Finally, the proteins were analyzed by western blotting.

### Chromatin immunoprecipitation (ChIP)

Each individual cell line was grown to a density of 6–8 × 10^5^ cells/mL and 7 × 10^5^ cells were collected. The cells used for *UCP1* ChIP were first treated with 100 nM human recombinant IRISIN (# SRP8039, Sigma) for 7 days at 37 °C, 5% CO_2_. After stimulation, cells were cross-linked in 1% formaldehyde for 10 min at room temperature and lysed by sonication to shear the DNA to fragments between 200 and 1000 base pairs, ensuring that the samples were kept cold (100% duty cycle for 7 × 30-s intervals). Lysates were treated overnight at 4 °C with 8 μg of UCP1 antibody (ab209483, Abcam). Protein-DNA complexes were captured on Dynabeads-Protein G (cat #, Millipore) and eluted in 1% SDS TE buffer at 65 °C. The pellets were resuspended in the appropriate buffer for PCR (95 °C 5 min, 95 °C 30 s, 56 °C 30 s, 72 °C 10 s, 72 °C 5 min, 4 °C 60 min, cycles 30) (TSE006, 2× T5 Super PCR Mix).

### Luciferase reporter gene assay

After cloning and amplifying the *UCP1* promoter region, the PCR products were cloned into the multiple cloning sites of pGL4.10. HEK-293T cells were co-transfected with pGL4.10-*UCP1*-prom- oter, PGL4.74 (Promega) and pReceiver-M02 (Genecopoeia) using Lipo3000 Transfection Reagent (Thermo Fisher Scientific, USA). After 48 h, cells were lysed, 100 μL of the cell lysate was added to each well, and the cells were incubated on a shaker at room temperature (24–26 °C) for 15 min. The cell lysate (20 μL) was added to 100 μL of luciferase assay reagent II (LARII) automatically by the machine. Next, 100 μL of Renilla luciferase assay reagent was added to the mixture to determine Renilla luciferase activity. The ratio of firefly luciferase and TK Renilla luciferase activity represented the reporter gene activity value. TK Renilla luminescence value was used as an internal reference. The experiment was performed in triplicate and expressed as the mean ± SD.

### Biofilm interference (BLI)

BLI is a type of surface reaction that detects the displacement changes in the interference spectrum. Two beams of optical film at the end of the sensor form two reflection spectra when a beam of visible light is emitted from the spectrometer. The interface forms a beam of interference for any shape due to molecular binding or dissociation. The thickness and density of the film can be reflected by the displacement value of the interference spectrum and pass through this displacement. The values were analyzed in real time. The APS was coupled and solidified. The coupled sample was IRISIN protein with a solidification concentration of 10 μg/ mL, and the dilution buffer was PBS (pH 7.4). The analyte sample was TRPC3 protein (provided by Chen Lei Laboratory, Peking University), and the dilution buffer used was PBST (pH 7.4, 0.05% Tween20). Analyte concentrations ranged from 500 nmol/L, 250 nmol/L, 125 nmol/L, 62.5 nmol/L, 31.25 nmol/L, 15.63 nmol/L, to 0 nmol/L. The reference buffer was PBST + 5% DMSO (pH 7.4 PBS, Tween20 0.05% + 5% DMSO, v/v). The experiment was performed at 1000*g*, 30 °C, using 220 μL/well. The experimental data were processed using Data Analysis software 9.0.

#### Statistical analysis

All statistical analyses were performed using SPSS 17.0 software. The data are expressed as mean ± standard deviation (*x* ± *s*). We used an independent group *t*-test to compare the means between two groups. Statistical significance among multiple groups was analyzed by one-way ANOVA (GraphPad Software, Inc). *, **, and *** indicates *p* < 0.05, *p* <0.01, and *p* < 0.001 statistical differences compared to control, respectively (*n* ≥ 3). Western blot analysis was performed using the AlphaView SA software for grayscale analysis to evaluate relative expression.

## Supplementary Information

**Additional file 1: Figure S1.** The detection of cellular Ca^2+^ by the Si NWs sensors.

**Additional file 2: Figure S2.** Detection of the interaction between IRISIN and TRPC3 in 293T cells.

**Additional file 3: Figure S3.** Intracellular Ca^2+^ levels increase upon IRISIN stimulation.

**Additional file 4: Figure S4.** *TRPC3* siRNA transfection downregulates the beige fat marker gene and reduces calcium ion influx.

**Additional file 5: Figure S5.** IRISIN mediates white fat beiging in mice.

**Additional file 6: Table S1.** Mass spectrometry of protein complexes immunoprecipitated with IRISIN.

## Data Availability

All data generated or analyzed during this study are included in this published article, its supplementary information files and publicly available repositories.

## Abbreviations

MSCs: Mesenchymal stem cells
T2D: Type 2 diabetes
UCP1: The uncoupling protein 1
WAT: White adipose tissue
FNDC5: Fibronectin III structure containing 5
PGC1-α: PPARγ-coactivator 1α
MAPK: The p38 mitogen-activated protein kinase
ERK: Extracellular-signal regulated kinase
SOCs: Store-operated Ca2+ channels
TRPC3/6/7: Transient receptor potential channel, canonical 3/6/7
AD-MSCs: Adipose tissue-derived MSCs
OCR: The oxygen consumption rate
SiRNA: Small interfering RNA
p-PPARγ: The phosphorylated PPARγ
PLC-gamma1: Phospholipase C gamma1
BLI: Biofilm interference
H-DMEM: High glucose
ChIP: Chromatin immunoprecipitation
