# Supplementary figures and images for: Irisin mediates beiging of adipose-derived mesenchymal stem cells through binding to TRPC3
Source: BMC Biol. 2022 May 2;20:95. doi: 10.1186/s12915-022-01287-2 (PMC9063202; doi:10.1186/s12915-022-01287-2)

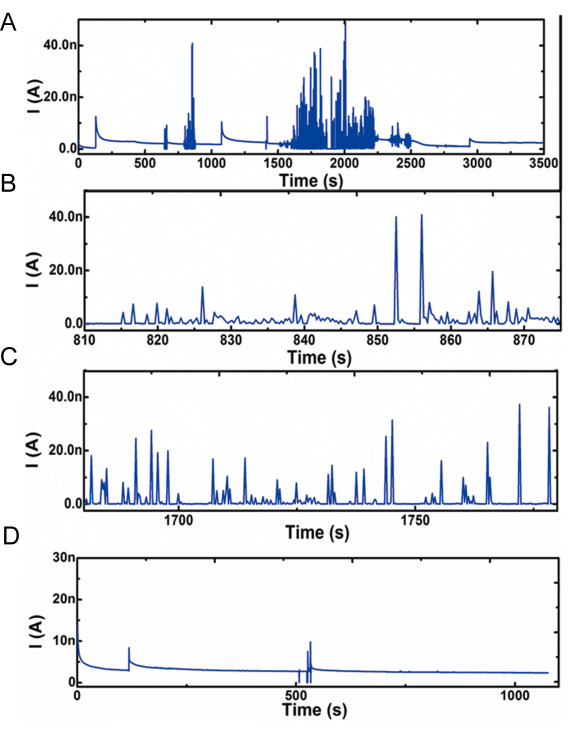

Supplement: Supplementary file 1 — Additional file 1: Figure S1. The detection of cellular Ca2+ by the Si NWs sensors. [file 12915_2022_1287_MOESM1_ESM.tif]

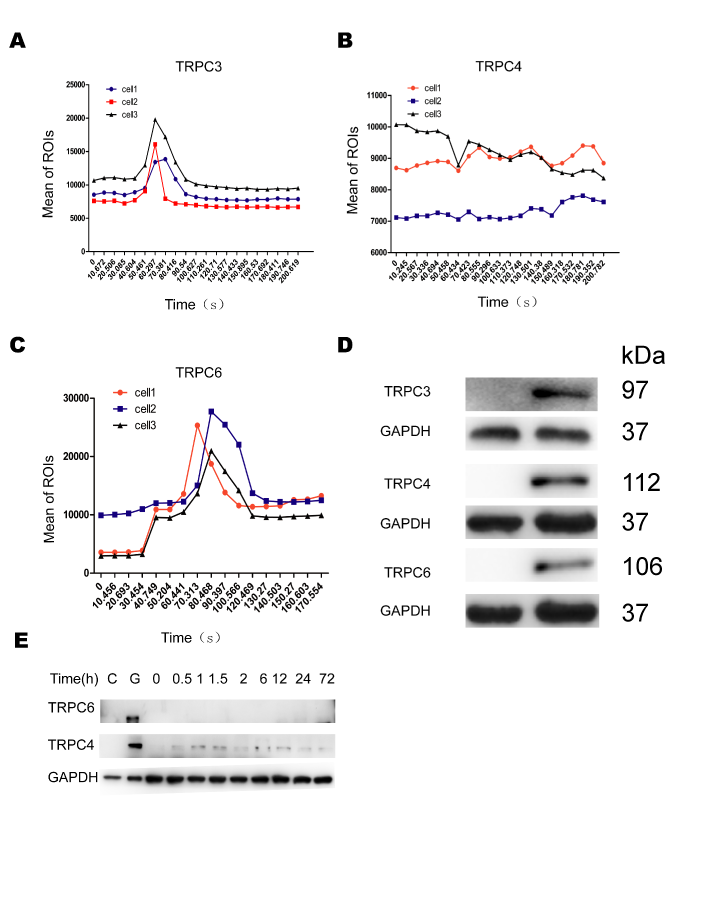

Supplement: Supplementary file 2 — Additional file 2: Figure S2. Detection of the interaction between IRISIN and TRPC3 in 293T cells. [file 12915_2022_1287_MOESM2_ESM.tif]

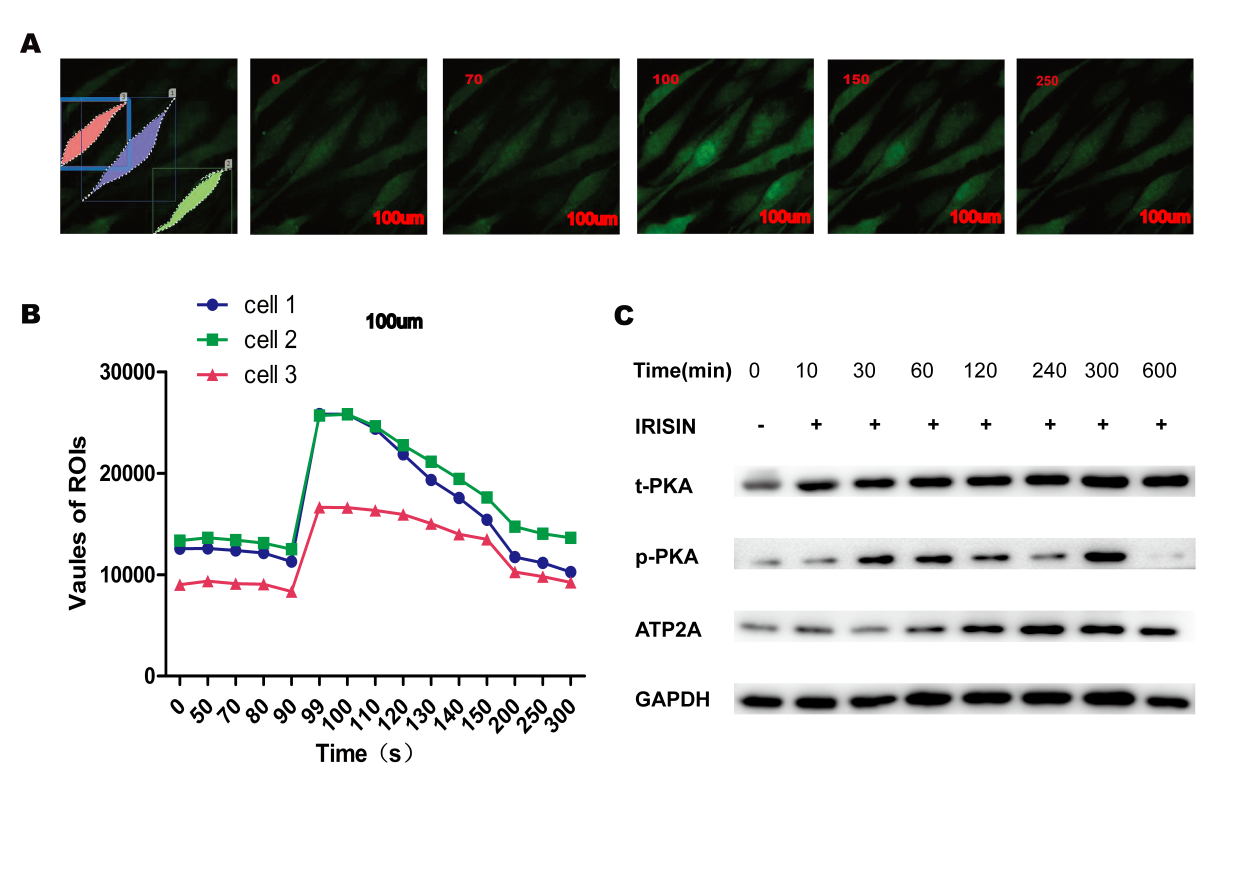

Supplement: Supplementary file 3 — Additional file 3: Figure S3. Intracellular Ca2+ levels increase upon IRISIN stimulation. [file 12915_2022_1287_MOESM3_ESM.tif]

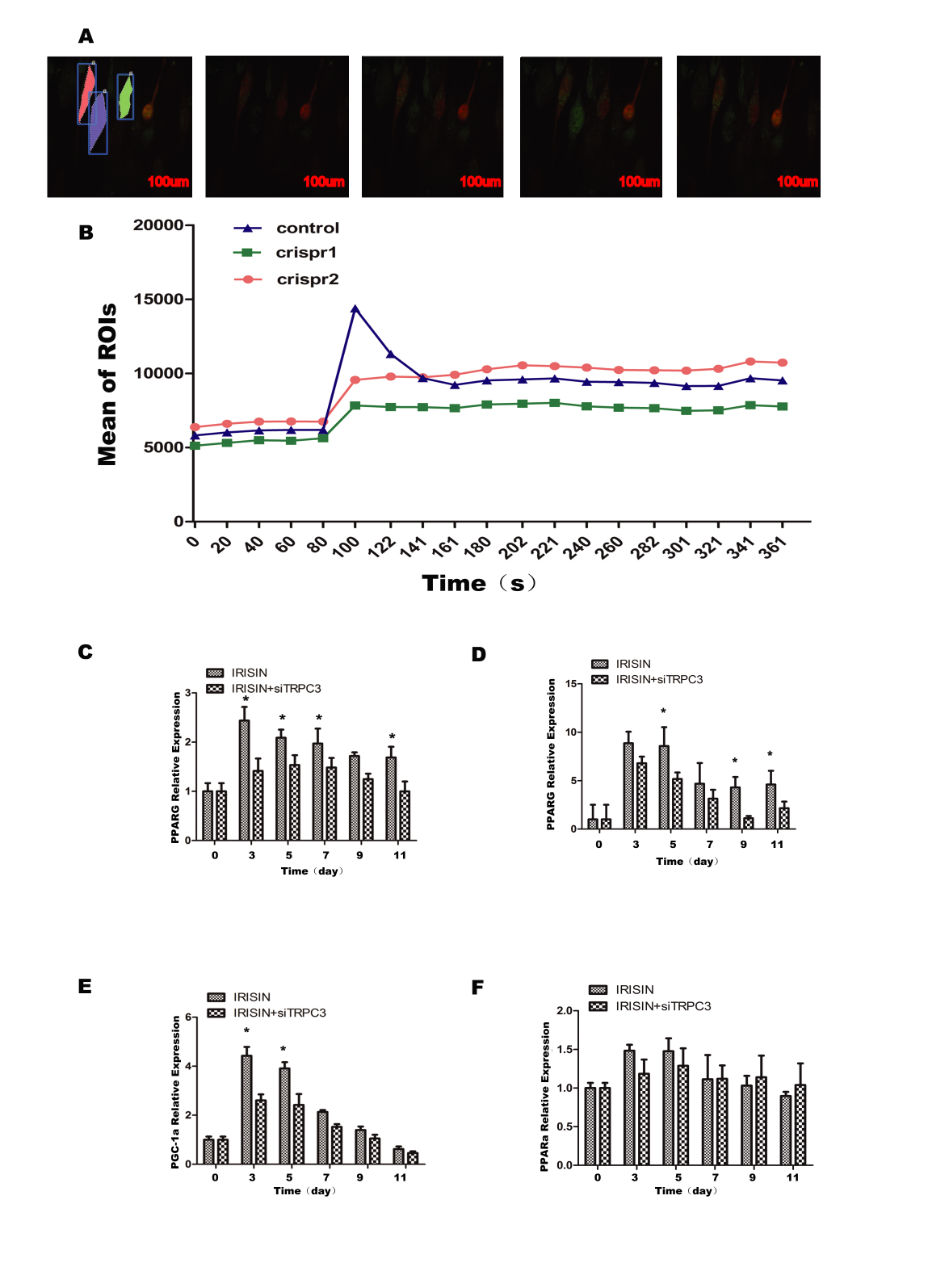

Supplement: Supplementary file 4 — Additional file 4: Figure S4. TRPC3 siRNA transfection downregulates the beige fat marker gene and reduces calcium ion influx. [file 12915_2022_1287_MOESM4_ESM.tif]

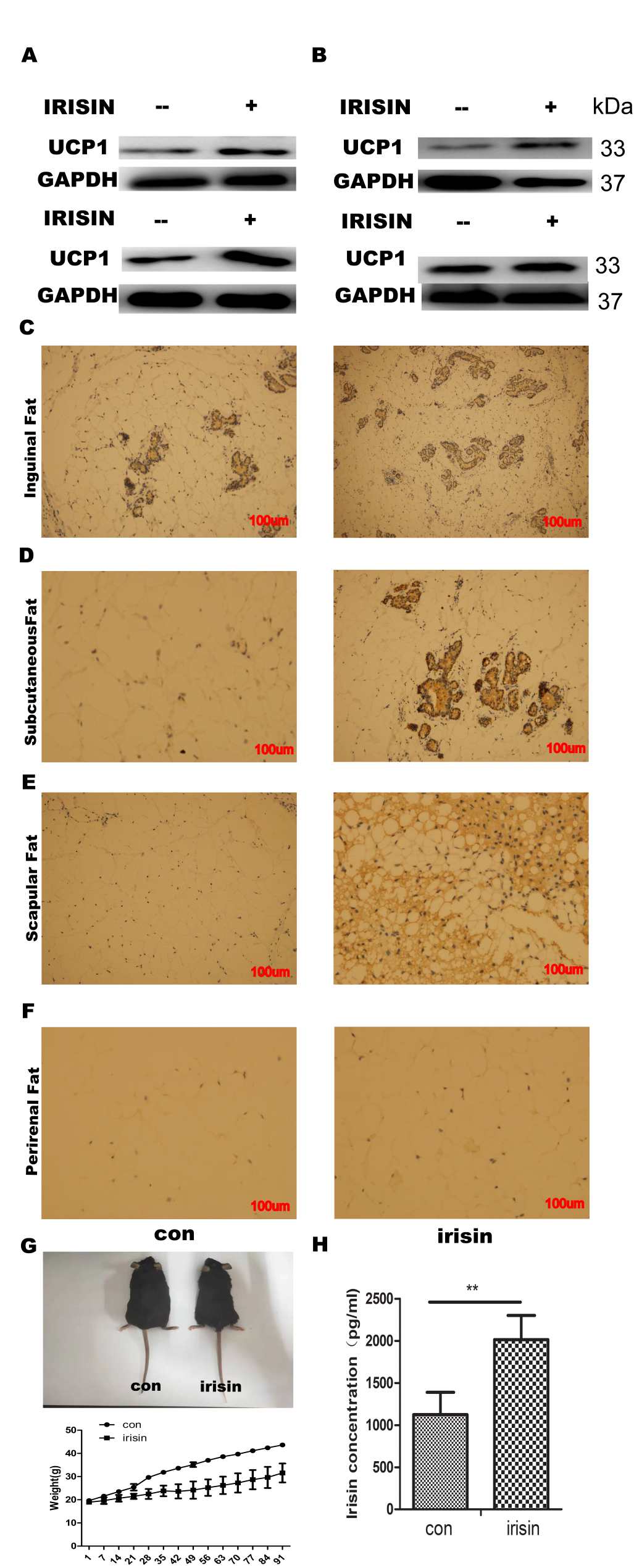

Supplement: Supplementary file 5 — Additional file 5: Figure S5. IRISIN mediates white fat beiging in mice. [file 12915_2022_1287_MOESM5_ESM.tif]
