# Supplementary material for: Irisin mediates beiging of adipose-derived mesenchymal stem cells through binding to TRPC3
Source: BMC Biol. 2022 May 2;20:95. doi: 10.1186/s12915-022-01287-2 (PMC9063202; doi:10.1186/s12915-022-01287-2)
Supplement: Supplementary file 6 — Additional file 6: Table S1. Mass spectrometry of protein complexes immunoprecipitated with IRISIN. [file 12915_2022_1287_MOESM6_ESM.pdf]

N	Accession	Name	Species	Peptides(95%)
1	sp|P35527|K1C9_HUMAN	Keratin, type I cytoskeletal 9 OS=Homo sapiens GN=KRT9 PE=1	HUMAN	2
2	sp|P13645|K1C10_HUMAN	Keratin, type I cytoskeletal 10 OS=Homo sapiens GN=KRT10 PE	HUMAN	2
3	sp|Q13507-2|TRPC3_HUMAN	Isoform 2 of Short transient receptor potential channel 3 O	HUMAN	3
4	sp|P35908|K22E_HUMAN	Isoform 2 of Short transient receptor potential channel 3 O	HUMAN	1
5	tr|Q9BZ22|Q9BZ22_HUMAN	Isoform 2 of Short transient receptor potential channel 3 O	HUMAN	1
6	tr|D6RGX4|D6RGX4_HUMAN	Protein FAM90A26P OS=Homo sapiens GN=FAM90A26P PE=4 SV=1	HUMAN	1
7	sp|Q86YD7|F90A1_HUMAN	Protein FAM90A1 OS=Homo sapiens GN=FAM90A1 PE=2 SV=3	HUMAN	1
8	sp|Q658T7|F90A2_HUMAN	Putative protein FAM90A2P OS=Homo sapiens GN=FAM90A2P PE=5	HUMAN	1
9	sp|Q658T7-2|F90A2_HUMAN	Isoform 2 of Putative protein FAM90A2P OS=Homo sapiens GN=F	HUMAN	1
10	sp|P0C7X0|F90AO_HUMAN	Putative protein FAM90A24P OS=Homo sapiens GN=FAM90A24P PE=	HUMAN	1
11	sp|P0C7W9|F90AE_HUMAN	Putative protein FAM90A14P OS=Homo sapiens GN=FAM90A14P PE=	HUMAN	1
12	sp|P0C7W8|F90AD_HUMAN	Putative protein FAM90A13P OS=Homo sapiens GN=FAM90A13P PE=	HUMAN	1
13	sp|P0C7V4|F90AJ_HUMAN	Putative protein FAM90A15P OS=Homo sapiens GN=FAM90A15P PE=	HUMAN	1
14	sp|A8MXZ1|F90AN_HUMAN	Putative protein FAM90A23P OS=Homo sapiens GN=FAM90A23P PE=	HUMAN	1
15	sp|A8MXJ8|F90A5_HUMAN	Putative protein FAM90A5P OS=Homo sapiens GN=FAM90A5P PE=5	HUMAN	1
16	sp|A8MX19|F90AC_HUMAN	Putative protein FAM90A12P OS=Homo sapiens GN=FAM90A12P PE=	HUMAN	1
17	sp|A8MWA6|F90AM_HUMAN	Putative protein FAM90A22P OS=Homo sapiens GN=FAM90A22P PE=	HUMAN	1
18	sp|A6NNJ1|F90A9_HUMAN	Putative protein FAM90A9P OS=Homo sapiens GN=FAM90A9P PE=5	HUMAN	1
19	sp|A6NKC0|F90A7_HUMAN	Putative protein FAM90A7P OS=Homo sapiens GN=FAM90A7P PE=5	HUMAN	1
20	sp|A6NJQ4|F90A8_HUMAN	Putative protein FAM90A8P OS=Homo sapiens GN=FAM90A8P PE=5	HUMAN	1
21	sp|A6NIJ5|F90AK_HUMAN	Putative protein FAM90A20P OS=Homo sapiens GN=FAM90A20P PE=	HUMAN	1
22	sp|A6NEW6|F90AG_HUMAN	Putative protein FAM90A16P/FAM90A17P OS=Homo sapiens GN=FAM	HUMAN	1
23	sp|A6NE21|F90AI_HUMAN	Putative protein FAM90A18P/FAM90A19P OS=Homo sapiens GN=FAM	HUMAN	1
24	sp|A6NDY2|F90AA_HUMAN	Putative protein FAM90A10P OS=Homo sapiens GN=FAM90A10P PE=	HUMAN	1
25	sp|Q9BQ51|PD1L2_HUMAN	Programmed cell death 1 ligand 2 OS=Homo sapiens GN=PDCD1LG	HUMAN	1
26	sp|Q9BQ51-2|PD1L2_HUMAN	Isoform 2 of Programmed cell death 1 ligand 2 OS=Homo sapie	HUMAN	1
27	sp|P40197|GPV_HUMAN	Platelet glycoprotein V OS=Homo sapiens GN=GP5 PE=1 SV=1	HUMAN	1
28	tr|B7Z8R6|B7Z8R6_HUMAN	Trypstatin OS=Homo sapiens GN=AMBP PE=2 SV=1	HUMAN	1
29	sp|P02760|AMBP_HUMAN	Protein AMBP OS=Homo sapiens GN=AMBP PE=1 SV=1	HUMAN	1
30	tr|Q5JXX3|Q5JXX3_HUMAN	Mortality factor 4 like 2 (Fragment) OS=Homo sapiens GN=MOR	HUMAN	3
31	tr|H0YNX3|H0YNX3_HUMAN	Mortality factor 4-like protein 1 (Fragment) OS=Homo sapien	HUMAN	5
32	tr|H0YLJ3|H0YLJ3_HUMAN	Mortality factor 4-like protein 1 OS=Homo sapiens GN=MORF4L	HUMAN	4
33	sp|Q9UBU8-2|MO4L1_HUMAN	Isoform 2 of Mortality factor 4-like protein 1 OS=Homo sapi	HUMAN	1
34	sp|Q15014|MO4L2_HUMAN	Mortality factor 4-like protein 2 OS=Homo sapiens GN=MORF4L	HUMAN	1

35	sp|P35527|K1C9_HUMAN	Keratin, type I cytoskeletal 9 OS=Homo sapiens GN=KRT9 PE=1	HUMAN	3
36	sp|Q9NS23|RASF1_HUMAN	Ras association domain-containing protein 1 OS=Homo sapiens	HUMAN	1
37	sp|Q9NS23-5|RASF1_HUMAN	Isoform E of Ras association domain-containing protein 1 OS	HUMAN	4
38	tr|J3QLJ4|J3QLJ4_HUMAN	Uncharacterized protein OS=Homo sapiens PE=4 SV=1	HUMAN	1
